# Psychotropic drug-induced weight gain and untargeted metabolomics: machine learning-driven results from a prospective cohort study

**DOI:** 10.1038/s41398-026-04129-9

**Published:** 2026-06-27

**Authors:** Marianna Piras, Gaëlle Magliocco, Setareh Ranjbar, Sylvain Le Gludic, Marie Gasser, Franziska Gamma, Martin Preisig, Séverine Crettol, Frederik Vandenberghe, Nicolas Ansermot, Carole Grandjean, Céline Dubath, Armin von Gunten, Philippe Conus, Aurelien Thomas, Chin Bin Eap

**Affiliations:** 1 Unit of Pharmacogenetics and Clinical Psychopharmacology, Centre for Psychiatric Neuroscience, Department of Psychiatry, Lausanne University Hospital, University of Lausanne, Prilly, Switzerland; 2 Unit of Forensic Toxicology and Chemistry, CURML, Lausanne and Geneva University Hospitals, Lausanne, Geneva, Switzerland; 3 Center for Psychiatric Epidemiology and Psychopathology, Department of Psychiatry, Lausanne University Hospital, University of Lausanne, Prilly, Switzerland; 4 Les Toises Psychiatry and Psychotherapy Center, Lausanne, Switzerland; 5 Service of Old Age Psychiatry, Department of Psychiatry, Lausanne University Hospital, University of Lausanne, Prilly, Switzerland; 6 Service of General Psychiatry, Department of Psychiatry, Lausanne University Hospital, University of Lausanne, Prilly, Switzerland; 7 Faculty Unit of Toxicology, CURML, Faculty of Biology and Medicine, University of Lausanne, Lausanne, Switzerland; 8 University of Lausanne, Lausanne, Switzerland

**Keywords:** Physiology, Biomarkers

## Abstract

Several psychotropic drugs can induce important weight gain. The present study aims to elucidate the metabolomic fingerprint of this adverse effect. From the Psymetab cohort, 151 follow-ups from 135 patients starting a weight-inducing psychotropic drug were selected. Untargeted metabolomics analyses were applied to plasma samples collected at treatment baseline and at another time point during the follow-up. Dimension reduction and feature selection approaches such as Minimum-Redundancy/Maximum-Relevance coupled with a bootstrap of 5000 Least Absolute Shrinkage and Selection Operator were applied to select metabolic features associated with weight change per month of treatment. Our selection algorithm identified 56 metabolic features. Out of the 56, 10 were identified, of which 6 were confirmed by standard references. Consistent with previous studies, increases in the intensity changes of glycerophospholipids such as lysophosphatidylcholine (18:2/0:0), and tryptophan metabolites such as acetylkynurenine and 5-hydroxy-L-tryptophan were positively associated with weight change. Moreover, increases in the intensity changes of the amino acids citrulline and N-Acetyl-L-aspartic, and of the xanthine 1-methyluric acid and of the pyrimidine nucleoside uridine were positively associated with weight changes, whereas increases in the intensity changes of the amino acid glutamine and of the steroidal glycoside glycochenodeoxycholic acid 3-glucuronide were negatively associated with the outcome. The present study highlighted novel potential biomarkers for weight change under psychotropic treatment, confirming an alteration in the pathways of glycerophospholipids and tryptophan metabolites. These results emphasise the potential use of metabolomics for clarifying metabolic changes and for defining biomarkers with a prospective use in clinical practice.

## Introduction

Several first-line medications prescribed to treat severe mental illnesses, such as schizophrenia, bipolar disorders, and major depression, are known to induce weight gain, blood glucose and lipid dysregulations [1]. These metabolic adverse effects are risk factors for the development of cardiovascular diseases, which are highly prevalent among the psychiatric population and contribute to reducing their life expectancy versus the general population by >10 years [2]. The mechanisms leading to psychotropic-induced metabolic adverse effects are complex, with both central and peripheral pathways playing a role [3]. Indeed, psychotropic drugs can interfere with hypothalamic transmissions, notably by blocking the histamine 1 and serotonin 2 C receptors, leading to increased hunger and food intake [3]. Additionally, hyperprolactinemia due to the blockade of dopamine receptors in the pituitary gland, and psychotropic drugs’ interactions with pancreatic, hepatic, and skeletal cellular activity can also lead to metabolic disturbances, such as blood glucose and lipid dysregulations, even in the absence of weight gain [3]. Moreover, there is high inter-individual variability in psychotropic-induced metabolic adverse effects, which has led to the identification of several risk factors, including young age, non-smoking status, Caucasian ethnicity, and low baseline weight [4, 5]. Given their complex mechanisms, several approaches (e.g., genetic, epigenetic [6, 7]) to further understand psychotropic-induced adverse effects have been explored.

Metabolomic analyses are nowadays widely used to characterize novel biomarkers and to describe biological patterns [8]. Indeed, these analyses describe the downstream products of cellular activity, investigating a limited or a large-scale number of metabolites through targeted or untargeted approaches, respectively. The metabolomic fingerprint of psychotropic drug-induced metabolic adverse effects has been previously investigated using these two approaches. According to a targeted longitudinal analysis including 62 psychiatric patients starting a psychotropic drug, 3 months of weight gain was predicted by one month of modifications in the concentration of blood markers associated with weight gain, including kynurenine, hexanoylcarnitine and biliverdin [9]. On the other hand, in a 12-week longitudinal analysis including 17 patients with minimal exposure to psychotropic drugs (≤ 2 weeks in the last three months), a targeted analysis did not detect any statistically significant difference in the concentration of the pre-determined metabolites from baseline [10]. However, patients with a weight gain of ≥5% following treatment start presented increased baseline concentrations of the fatty acid adrenic acid versus the patients with a weight gain of <5% [10]. Moreover, among 27 drug-naive female patients taking the antipsychotic olanzapine for 4 weeks, an untargeted analysis showed an up-regulation of lysophosphatidylcholines (LysoPC) and lysophosphatidylethanolamines, and a down-regulation of carnitine after one month of treatment as compared to baseline [11].

Given the limited evidence describing biomarkers and their metabolic pathways associated with psychotropic-induced weight changes, the present study aims to conduct a longitudinal investigation of the metabolomic changes among a Swiss cohort of psychiatric patients starting the intake of weight-inducing psychotropic drugs.

## Methods

### Study subjects and samples

Data and samples for 135 patients recruited between 2013 and 2022 come from the Psymetab cohort, a study approved by the Canton of Vaud (protocol ID: 2017-01301) and started in 2007 at Lausanne University Hospital in collaboration with a private healthcare center Les Toises, Lausanne, for which patients signed an informed consent. Patients were included in the present study if they were starting a psychotropic drug known to potentially induce important weight gain (see Supplementary Table 1 for the drug list), and if blood samples were collected at treatment baseline (T0) and at one other time point during clinical routine controls (TX, i.e., from one month to one year of treatment). Of note, whether patients were psychotropic-drug naive or were switching to a new psychotropic drug could not be assessed. To ascertain fasting conditions, only blood samples collected during a hospital stay were included. Of the 135 patients, 16 had T0 and TX blood samples for two different follow-ups (i.e., starting of two different weight-inducing psychotropic drugs). Thus, a total of 151 independent follow-ups were included in the present study (i.e., 119 patients with one single follow-up plus 16 patients with 2 follow-ups but different medications, thus considered as independent follow-ups). High sensitivity CRP levels (Cobas integra 400 plus Roche®, Roche Diagnostic, Rotkreuz, Switzerland) were also analyzed at T0 and TX for each follow-up.

### Untargeted metabolomic analysis

Plasma samples were collected in a hospital environment and kept at −80 °C until analysis.

#### Metabolite extraction

100 μL of plasma sample were diluted in 400 μL of mix solvent EtOH-MeOH 1:1 containing 10 ng/mL of internal standards hydrocodone-d6 and phenobarbital-d5. Samples were vortexed and centrifuged for 20 min at 8 °C and 13,000 rpm. Supernatants were recovered and evaporated for 3 h at room temperature with a CentriVap Concentrator (Labconco, Kansas City, MO, USA). Samples were stored at −80 °C before further preparation.

#### Sample preparation

The pellets were resuspended in 100 μL of 10% MeOH, vortexed for 15 s and centrifuged for 20 min at 8 °C and 13,000 rpm. The supernatants were divided into two fractions of 40 μL and were transferred into two glass vials with inserts for further injection in positive and negative modes. Quality control samples were generated by combining 10 μL of each experimental sample to form a pooled matrix sample.

#### Untargeted mass spectrometry

Metabolite samples were analyzed by ultrahigh performance liquid chromatography (Ultimate 3000 LC system, Thermo Fisher Scientific) coupled to high-resolution mass spectrometry (Orbitrap Exploris 120, Thermo Fisher Scientific) interfaced with a heated electrospray ionization (HESI-II). Positive and negative mode mobile phases A and B consisted of 0.1% formic acid in water and 0.1% formic acid in methanol respectively. The sample injection volume was 5 μL and separation was made using a C18 column (Phenomenex Kinetex, C18 – 50 × 2.1 mm, 2.6 um) held at 40 °C. Mobile phase B was ramped linearly from 2–98% over 6 min, reached 100% at the 9^th^ min and was brought back to 2% for a 4 min re-equilibration stage. The total analysis run was 13 min with a flow of 300 μL/min. The mass analyzer was calibrated for mass accuracy and the full scan range covered 70–1000 m/z. The ionization spray voltage was set to 3.9 and 3.2 kV and the sheath gas flowrate was set to 25 and 30 (in arbitrary units) in positive and negative mode, respectively. The auxiliary gas flowrate was set to 5 in both modes (in arbitrary units). The acquisition software was TraceFinder 5.1 (Thermo Fisher Scientific). Before analysis, the samples were randomized to avoid any biological and analytical bias. They were then divided into four batches, corresponding to four independent LC–MS runs performed on seperate days. Within each batch, pooled QC samples were injected regularly throughout the run to monitor analytical stability. More specifically, QC samples were injected at the beginning of the batch and subsequently at regular intervals (one QC every 7samples).

#### Data analysis

Raw metabolomics data were uploaded to MS-Dial software (version 4.9.221218) for peak detection, chromatogram alignment, missing data estimation and blank subtraction [12]. Data were normalized in MS-Dial by LOWESS normalization with a span threshold of 0.8.

### Metabolite identification

MS/MS scanning was carried out using the full-scan/dd-MS2 mode on the instrument and the chromatographic conditions described above. The full scan range covered 70–1000 m/z with resolution at 60,000. When the intensities of the precursors on the inclusion list were above the intensity threshold of 2.0e5cps, MS^2^ mode was triggered. The precursor was selected in the quadrupole with an isolation window of 1 Da. Fragmentation was performed with HCD collision energies of 15, 30 and 45%. All fragments were acquired as centroid data with a resolution of 15,000.

Structural identification was then performed using Compound Discoverer 3.3 (Thermo Fisher Scientific), SIRIUS 6.0.0, MzCloud (http://www.mzcloud.org) and HMDB (http://www.hmdb.ca/). These databases were used to help identify the molecular structures of the selected features based on the available experimental data (i.e., exact MWs, molecular formulas and fragmentation patterns).

According to the identification degree of confidence, metabolites were classified into four different categories according to the Chemical Analysis Working Group, Metabolomics Standards Initiative (MSI) [13]. Level I includes metabolites whose identification was confirmed using reference standards (i.e., matching of m/z, RT and MS/MS); level II includes metabolites putatively annotated after checking similarity with spectral libraries (i.e., matching of m/z and/or MS/MS); level III includes putative compound classes; level IV includes unknown metabolites.

Glucuronidated compounds were identified through neutral loss of the glucuronic acid moiety (−176 m/z) and by matching them to standards of the corresponding deglucuronidated compounds, as no direct standards for the glucuronidated forms were available. Since no standard was available for acetylkynurenine, we putatively identified this compound through the fragmentation pattern of the kynurenine standard. For both acetylkynurenine and the glucuronide compounds, a maximum identification confidence level of II was assigned where a convincing match was observed.

### Statistical analysis

Descriptive statistics were provided to describe the cohort and patients’ weight was compared between T0 and TX using the signed-rank Wilcoxon test. The selection of metabolic features associated with our outcome (i.e., weight change from baseline /per month of treatment) was applied for the intensity change from baseline of positively (POS) and negatively (NEG) charged features enhanced from the untargeted metabolomic analysis. The algorithm used for this selection is a hybrid type method that encompasses three different steps using filter-based methods and machine learning techniques or embedded-based methods [14]. First, features were filtered using quality control information provided, and those with a coefficient of variation higher than 20% in the quality control samples were discarded. Then, the Minimum Redundancy Maximum Relevance (mRMR) algorithm [15] with a threshold at 100 metabolic features was applied to the delta of features intensity per month of treatment (i.e., (intensity of metabolic features at TX-intensity T0)/month of treatment). Briefly, the mRMR filtering was done to select and save 100 POS and 100 NEG metabolic features that have the least redundancy among them and are the most relevant to the outcome of interest. At each iteration, the mRMR scores are defined as the Spearman Correlation Quotient (SCQ) [16], which is the mutual information between the outcome and the metabolic features divided by the average of mutual information of previously selected features. The metabolic feature with the maximum SCQ score is the next selected one on the 100-metabolite list. Supplementary Fig. 1 shows a plateau in the SCQ scores for the 51–100 selected metabolic features, supporting our decision to include not more than 100 features in our algorithm to avoid redundant results. The 100 highest SCQ scores are reported for the metabolic features included in the next step. On the resulting 100 POS and NEG metabolic features we performed Least Absolute Shrinkage and Selection Operator (LASSO) from elastic net [17] to further select features that enhance accuracy in the prediction of our outcome. To ensure stability, 5000 LASSO bootstrap repetitions were run, and the metabolic features that were selected at least 50% of the time (i.e., at least in 2500 bootstraps) and with a stable sign of their coefficients (i.e. the sign of their coefficient did not switch sides in different bootstrap repetitions) within the 95% empirical confidence interval were selected. The percentage of the time each of the selected feature were chosen by the LASSO algorithm is reported. In each of the 5000 rounds of the bootstrap, a 10-fold cross-validation was used to choose the optimum tuning parameter in the LASSO algorithm. Of note, in order to further validate our 100-metabolite mRMR threshold, additional tests performing 5000 LASSO bootstraps to 50 and 150 mRMR thresholds were evaluated, with the 100 mRMR threshold resulting in more stable and less redundant results (data not shown). Supplementary Fig. 2 shows the complete flowchart of the feature selection and Supplementary Table 2 shows the algorithm in a mathematical setting. A post-hoc volcano plot was generated to illustrate the univariate associations between the selected metabolic features and weight change per month, with models controlled for sex, age and smoking status and *p*-values adjusted for false discovery rate (FDR). In order to show the variability of the selected sets of metabolites (POS and NEG) in the 5000 LASSO bootstraps, the list of metabolites selected in one of the random LASSO iterations only was also reported.

## Results

Table 1 summarizes the demographic characteristics of our cohort reporting a median age of 43 years, an equal repartition between male (52%) and female sexes (48%), and a median overweight (i.e., ≥25 kg/m^2^) body mass index (BMI) at baseline. With a median treatment duration of one month, a significant increased weight was found among the 151 follow-ups at TX (*p* < 0.001), with no significant changes for CRP levels (*p* = 0.31).

**Table 1:** Demographics of included psychiatric follow-ups.

Variable^a^	*N* = 151
**Age (years)**^**b**^	43 (33 – 59)
**Females**	73 (48%)
**Diagnosis**^**c**^	
Schizophrenia or Schizoaffective disorder	85 (56%)
Bipolar disorder or Major depression	42 (28%)
Other	24 (16%)
**Smokers**	85 (56%)
Missing	1 (0.7%)
**Follow-up treatment duration (days)**^**d**^	33 (29 – 44)
**Weight at baseline (kg)**	71.7 (62 – 83)
**BMI at baseline (kg/m**^2^**)**	25.0 (21.3-29.4)
**Weight at second time point (kg)**	74 (65 – 85)
**BMI at second time point (kg/m**^2^**)**	25.9 (22.2 – 29.8)
**Weight change per month (%)**^**e**^	1.8 (0 – 4.7)
**Main psychotropic drugs**^f^	
Second-generation antipsychotics	102 (67.6%)
First-generation antipsychotics	21 (13.9%)
Mood stabilizers	21 (13.9%)
Antidepressants	7 (4.6%)
**Psychotropic co-medication**^g^	85 (56%)
**Antidiabetic prescription**^h^	11 (7.3%)
**Antihypertensive prescription**^h^	28 (18.5%)
**Lipid-lowering prescription**^h^	14 (9.3%)
**CRP levels at baseline (mg/l)**	1.5 (0.5 – 4.2)
**CRP levels at second time point (mg/l)**	1.3 (0.5 – 3.2)

^a^Continuous variables are reported as median (quartile 1 and 3) and categorical variables as number and percentage (%).

^b^Age range 18–93.

^c^International Classification of Diseases-10: organic disorders, anxiety, personality disorder, intellectual disability, dementia, and substance use disorder were classified together as “other.”

^d^Minimum and maximum values of treatment duration: 17 and 509 days, respectively.

^e^Mean weight change: 2.9%; standard deviation: 5.9%; minimum and maximum values: −14.3 and 21.5, respectively.

^f^Second-generation antipsychotics: amisulpride (*N* = 11); aripiprazole (*N* = 20); brexpiprazole (*N* = 2); cariprazine (*N* = 1); clozapine (*N* = 7); lurasidone (*N* = 6); olanzapine (*N* = 12); paliperidone (*N* = 4); quetiapine (*N* = 17); risperidone (*N* = 22). First-generation antipsychotics: haloperidol (*N* = 16); zuclopenthixol (*N* = 5). Mood stabilizers: lithium (*N* = 11); valproate (*N* = 10). Antidepressants: mirtazapine (*N* = 7).

^g^Weight-inducing psychotropic co-medications among drugs reported in Supplementary Table 1 at either T0 or TX. A total of 47, 18, 17, and 3 patients were receiving a second psychotropic medication among those taking a second-generation, first-generation antipsychotic, mood stabilizer and antidepressant as a main psychotropic drug, respectively.

^h^See Supplementary Table 1.

**Fig. 1:**
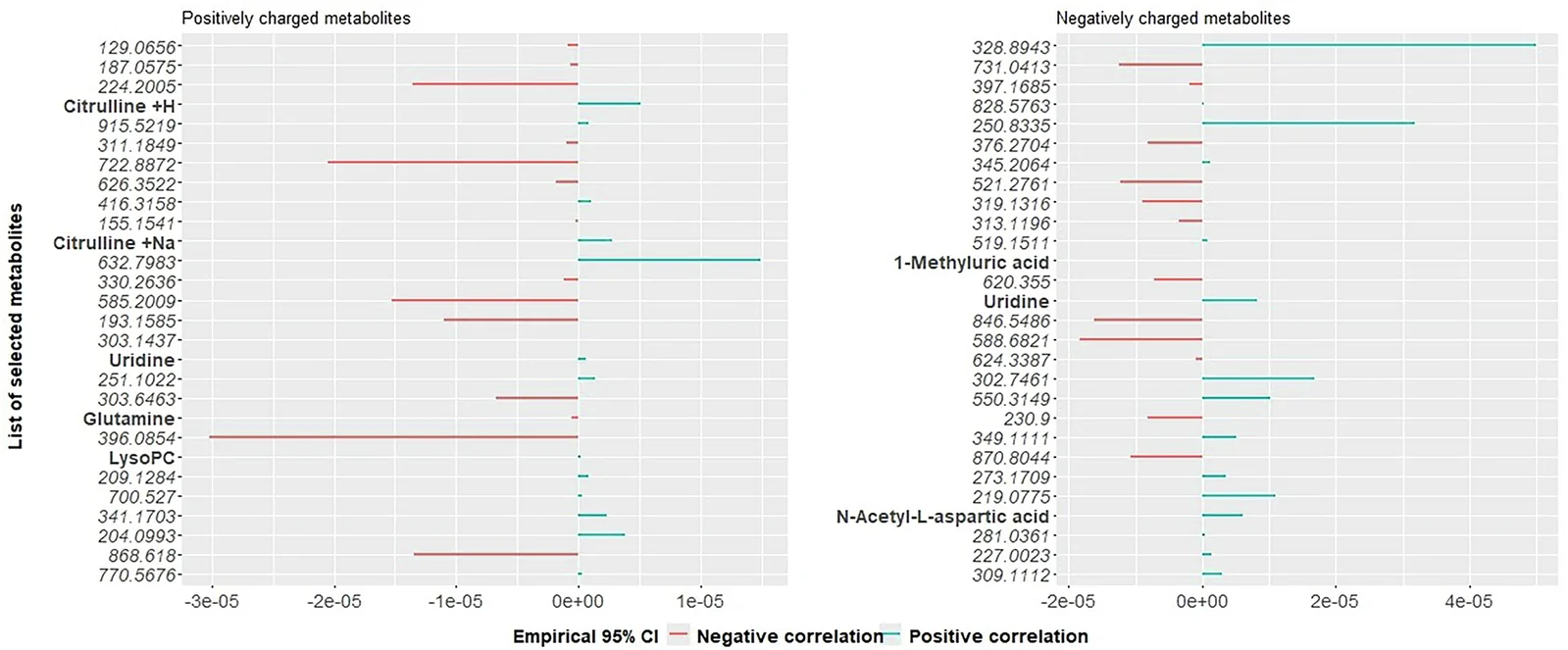
Association between positively and negatively charged metabolites, and weight change per month. Bars on the right or on the left indicate a positive or negative association between the delta intensity of the metabolites and weight change per month, respectively. The length of the bars represents the 95% empirical confidence interval from the 5000 bootstraps. Metabolites are ordered and plotted based on the percentage of time they were selected in 5000 bootstrap repetitions from top to bottom in decreasing order. The metabolites’ name is reported for metabolites identified with Metabolomics Standards Initiative (MSI) level I.

LOWESS normalization effectively corrected batch effects, as evidenced by the overlap of batches in the PCA score plot after correction, in contrast to their separation before normalization **(**Supplementary Fig. 3**)** [18]. In addition, PCA scores plot revealed a tight clustering of QC samples, indicating high analytical quality. Before filtering (Supplementary Fig. 2), the untargeted metabolomic analysis detected a total of 4599 and 6689 metabolic features in negative (NEG) and positive (POS) ionisation mode, respectively. With the first filtering step, 660 NEG and 1076 POS features were excluded for having a coefficient of variation greater than 20% in quality control samples. Thus, mRMR analysis was performed on the remaining 3939 NEG and 5613 POS metabolic features, keeping the 100 features whose intensity change was most associated with weight change from baseline weight per month of treatment, and less associated with each other (Supplementary Table 3). Subsequently, a LASSO feature selection step was performed with 5000 bootstrap iterations, which selected 28 POS and 28 NEG metabolic features (Supplementary Table 4, Supplementary Fig. 4). In accordance with their median intensity change (i.e., median[intensity at TX – T0 intensity]), these metabolic features could be most abundant at T0 (i.e., intensity at T0 > intensity at TX) or at TX (i.e., intensity at T0 < intensity at TX). The direction of the association between metabolic feature intensity changes (POS and NEG) and the outcome is shown in Fig. 1.

**Fig. 2:**
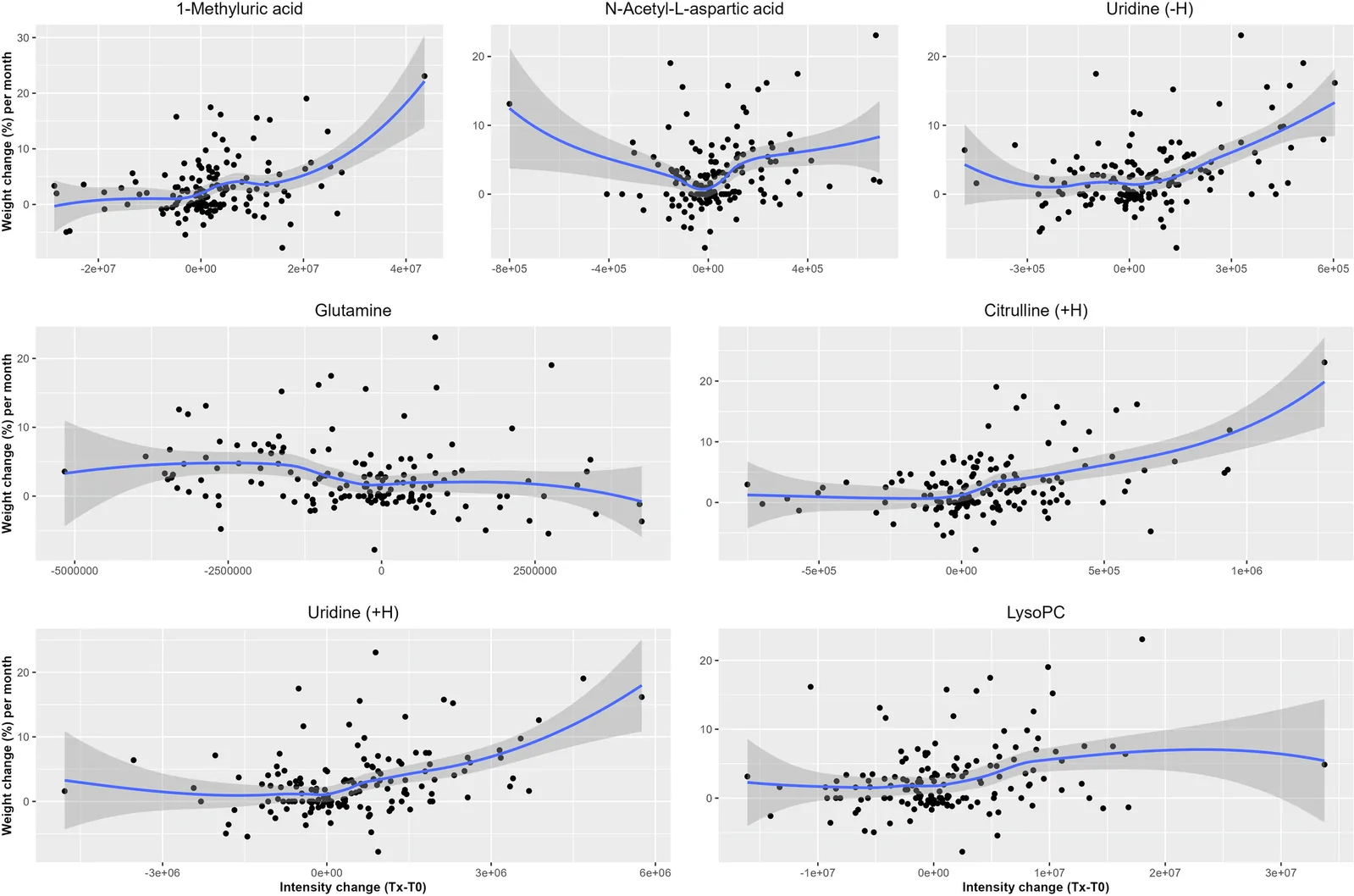
Intensity changes versus weight change for metabolites effectively identified (MSI level I). Weight change per month of psychotropic treatment versus intensity change (TX-T0) for metabolites identified with Metabolomics Standards Initiative (MSI) level I using smoothing spline with 95% confidence bands.

**Fig. 3:**
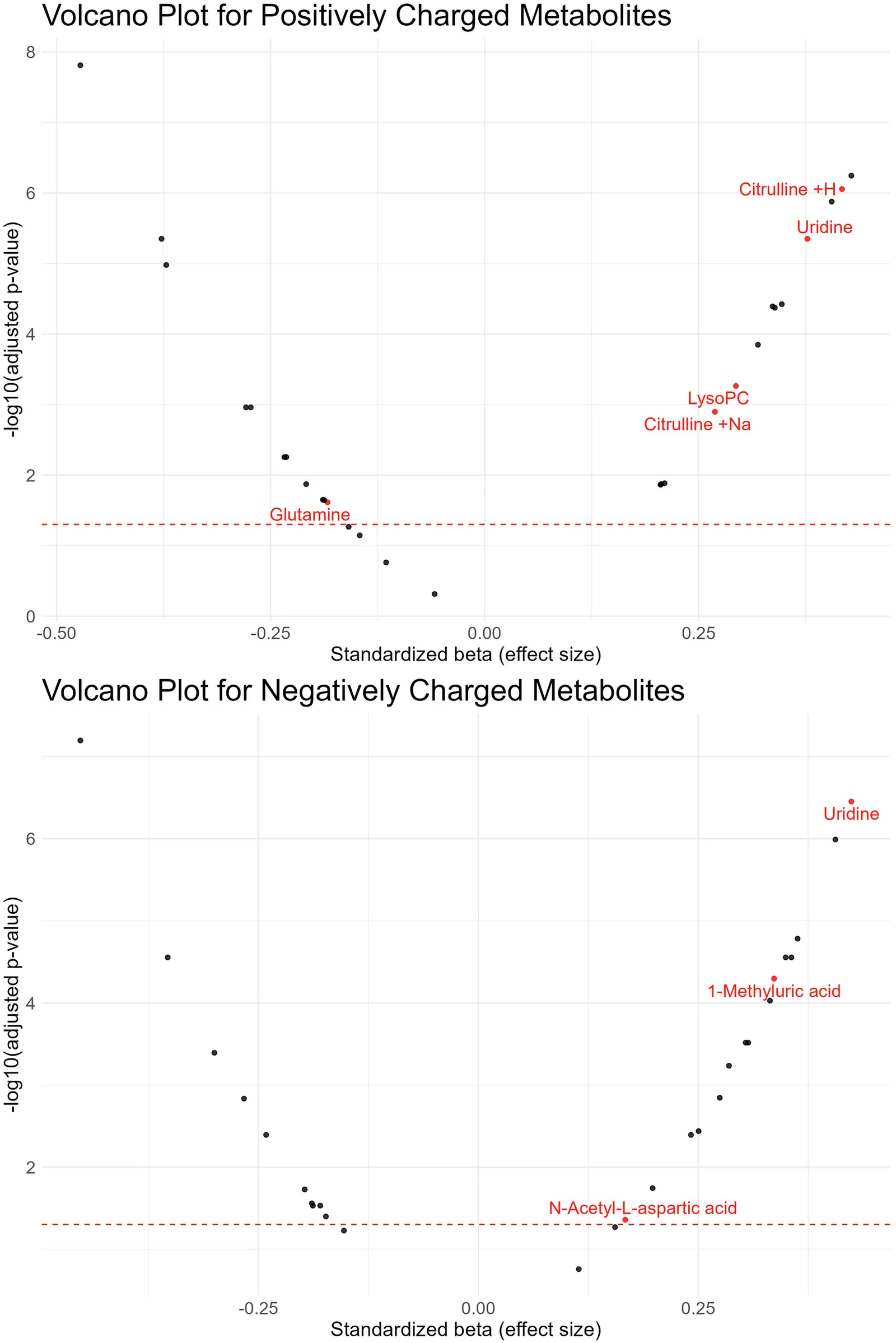
Volcano plot of the univariate association between positively and negatively charged metabolic features, and weight change per month after adjustment for age, sex and smoking status. Points above dashed lines represent the metabolic features that are significantly associated with weight change per month of treatment based on univariate regressions (i.e. one model per feature). Each model is adjusted for age, sex and smoking status. *P*-values were adjusted for multiple testing by false discovery rate among selected metabolic features. The metabolites’ names are reported for metabolites identified with Metabolomics Standards Initiative (MSI) level I. The x-axis represents the effect size in the form of Cohen’s d, where as y-axis is the -log of adjusted *p*-values.

The 28 POS and 28 NEG metabolic features selected using 5000 LASSO bootstraps were identified according to MSI criteria (Table 2; only levels of confidence I to III were included yielding 17 POS and 16 NEG). Notably, the metabolites glycochenodeoxycholic acid 3-glucuronide (GCDC3-glucuronide) and uridine were identified in both NEG and POS analysis. GCDC3-glucuronide was most abundant at T0, its intensity change being negatively associated with our outcome. Of note, although putatively identified, MS/MS spectra of GCDC3-glucuronide matched with the deglucuronidated GCDC3 standard. Uridine was most abundant at TX, and its intensity change was positively associated with our outcome. Among the other NEG identified compounds most abundant at TX, the xanthine 1-methyluric acid and the amino acid N-Acetyl-L-aspartic acid were identified with level I confidence, and the tryptamine 5-hydroxy-L-tryptophan was identified with level II. Concerning the POS compounds, on one hand, the amino acid glutamine (MSI level I), and the acylcarnitine 4,8 dimethylnonanoyl carnitine (MSI level II) were identified among the most abundant compounds at T0. On the other hand, the amino acids citrulline (MSI level I) and acetylkynurenine (MSI level II) and the LysoPC(18:2/0:0) (MSI level I) were identified among the most abundant compounds at TX. For metabolites identified with MSI level I, the intensity change (TX-T0) versus the weight change per month of psychotropic treatment is reported in Fig. 2 (see Supplementary Figs. 5 and 6 for all the LASSO-selected metabolites). As shown in the post-hoc volcano plot (Fig. 3), most metabolic features (and all metabolic features identified with MSI level I) showed significant associations with weight change per month, exceeding the threshold of –log(0.05), after adjusting for sex, age, and smoking status, with p-values corrected for false discovery rate. In order to show the variability of the selected sets of metabolites (POS and NEG), we also reported the list of metabolites selected in one of the LASSO iterations (see Supplementary Table 5).

**Table 2:** Metabolites identified in accordance with Metabolomics Standards Initiative criteria.

Sign	Most abundant	M/Z	RT	Adduct	Chemical formula	Putative name	Putative class#	Delta ppmL	MSI level	Identifier (Code HMDB / PUBMED)
NEG	T0	313.1196	6.0	M-H	C15H17F3N2O2	-	Amino acids and derivatives		3	
NEG	T0	870.8044	0.3	M-H	C28H14I2N2O11S2	-	1-naphthalene sulfonates		3	
NEG	T0	624.3387	5.6	M-H	C32H51NO11	Glycochenodeoxycholic acid 3-glucuronide	Steroidal glycosides	−0.37	2*	HMDB0002579
NEG	T0	345.2064	7.0	M-H	C21H30O4	-	Hydroxysteroids		3	
NEG	T0	846.5486	9.3	M-H	C44H82NO12P	-	Glycerophospholipids		3	
NEG	TX	181.0366	1.3	M-H	C6H6N4O3	1-Methyluric acid	Xanthines	−0.61	1	HMDB0003099
NEG	TX	328.8943	0.3	M-H	C8H5Cl3N2O4S	-	Benzenesulfonic acids and derivatives		3	
NEG	TX	174.0410	0.4	M-H	C6H9NO5	N-Acetyl-L-aspartic acid	N-acyl-L-alpha-amino acids	1.19	1	HMDB0000812
NEG	TX	273.1709	5.7	M-H	C14H26O5	-	Long-chain fatty acids		3	
NEG	TX	349.1111	5.4	M-H	C16H16F2N4O3	-	Pyridinecarboxylic acids		3	
NEG	TX	519.1511	4.4	M-H	C25H28O12	-	Phenolic glycosides		3	
NEG	TX	289.0680	0.7	M + FA-H	C9H12N2O6	Uridine	Pyrimidine nucleosides	1.04	1	HMDB0000296
NEG	TX	550.3149	6.4	M + FA-H	C25H48NO7P	-	Glycerophospholipids		3	
NEG	TX	309.1112	5.2	M-H	C14H18N2O6	-	Amino acids, peptides and analogues		3	
NEG	TX	828.5763	9.2	M-H	C45H84NO10P	-	Glycerophospholipids		3	
NEG	TX	219.0775	0.9	M-H	C11H12N2O3	5-hydroxy-L-tryptophan	Tryptamines and derivatives	−0.056	2	HMDB0000472
POS	T0	129.0656	0.4	M + H	C5H8N2O2	-	Alpha amino acids and derivatives		3	
POS	T0	147.0761	0.4	M + H	C5H10N2O3	Glutamine	D-alpha amino acids	−2.04	1	HMDB0000641
POS	T0	155.1541	2.5	M + H	C9H18N2	-	Cyclohexylamines		3	
POS	T0	187.0575	0.4	M + H	C12H10S	-	Diarylthioethers		3	
POS	T0	330.2636	5.8	M + H	C18H35NO4	4,8 Dimethylnonanoyl carnitine	Acyl carnitines	−0.87	2	HMDB0006202
POS	T0	626.3522	5.6	M + H	C32H51NO11	Glycochenodeoxycholic acid 3- glucuronide	Steroid glucuronide conjugates	−2.06	2*	HMDB0002579
POS	TX	176.1028	0.4	M + H	C6H13N3O3	Citrulline	Alpha amino acids	−0.97	1	HMDB0000904
POS	TX	198.0848	0.4	M+Na	C6H13N3O3	Citrulline	Alpha amino acids	−3.33	1	HMDB0000904
POS	TX	209.1284	4.0	M + H	C11H16N2O2	-	Alpha amino acids and derivatives		3	
POS	TX	251.1022	3.5	M + H	C12H14N2O4	Acetylkynurenine	N-acyl-alpha amino acids	−1.74	2*	HMDB0240342
POS	TX	267.0583	0.7	M+Na	C9H12N2O6	Uridine	Pyrimidine nucleosides	−3.76	1	HMDB0000296
POS	TX	303.1437	5.0	M + H	C14H22O7	-	Tricarboxylic acids and derivatives		3	
POS	TX	416.3158	4.3	M + H	C26H41NO3	-	Solanidine metabolite		3	,Magliocco et al.[50]
POS	TX	542.3214	7.0	M+Na	C26H50NO7P	LysoPC(18:2/0:0)	Lysophosphatidylcholines	−1.58	1	HMDB0010386
POS	TX	700.5270	9.2	M + H	C39H74NO7P	-	Glycerophospholipids		3	
POS	TX	770.5676	9.0	M + H	C43H80NO8P	-	Glycerophospholipids		3	
POS	TX	915.5219	6.5	M + H	C47H79O15P	-	Glycerophospholipids		3	

Metabolites were classified into four different categories according to the Chemical Analysis Working Group, Metabolomics Standards Initiative (MSI). Level I includes metabolites whose identification was confirmed using reference standards (i.e., matching of m/z, RT and MS/MS); level II includes metabolites putatively annotated after checking similarity with spectral libraries (i.e., matching of m/z and/or MS/MS); level III includes putative compound classes. Unknown metabolites (MSI level IV) were not included.

*NEG* negative charge, *POS* positive charge, *T0* treatment baseline, *TX* other time point during psychotropic treatment, *M* mass, *Z* charge, *HMDB* human metabolome database.

^#^Non-exhaustive list. For the exhaustive list see the HMDB website. *Match of MS/MS fragments with deglucuronidated/deacetylated standard.

## Discussion

With an untargeted metabolomic analysis and a machine learning approach, this study identifies 28 positively-charged and 28 negatively-charged metabolites predicting weight change per month of psychotropic treatment. Out of the 56, 10 metabolites involved in several physiological pathways were identified with a level-I (*N* = 6) and -II (*N* = 4) degree of confidence (see Table 3 for a summary). Among the features whose intensity changes were negatively associated with our outcome, we have found GCDC3-glucuronide, a steroidal glycoside synthesized in the liver by the Uridin-5’-diphospho (UDP)-glucuronosyltransferase, whose upregulation was previously associated with both reduced insulin sensitivity among men, and with type II diabetes [19, 20] (conditions often associated with an increase in weight [21]). Our results suggest that greater increases per month in the intensity levels of GCDC3-glucuronide are negatively associated with weight change during psychotropic treatment. The median overweight BMI at baseline in our population (see Table 1) might explain this result. Indeed, having an increased baseline weight was negatively associated with psychotropic-induced weight gain in previous studies [22, 23], suggesting a plateau effect in weight increase. Likewise, the small negative effect size association between our outcome and GCDC3-glucuronide intensity level changes, which may be indirectly associated with a T0 overweight status as previously mentioned, might suggest a plateau in psychotropic-induced weight. Nevertheless, further studies are needed to elucidate the role of GCDC3-glucuronide in the context of psychotropic-induced weight gain.

**Table 3:** Result summary of metabolites identified with level-I and -II degree of confidence.

Putative name	Sign	Most abundant	Main metabolic pathway(s) (HMDB)^#^	Association with weight change
1-Methyluric acid	NEG	TX	Caffeine Metabolism	Positive
4,8 Dimethylnonanoyl carnitine	POS	T0	Oxidation of Branched Chain Fatty Acids	Negative
5-hydroxy-L-tryptophan	NEG	TX	Tryptophan metabolism	Positive
Acetylkynurenine	POS	TX	NA	Positive
Citrulline	POS	TX	Arginine, Aspartate, Proline Metabolism Urea cycle	Positive
Glutamine	POS	T0	Arginine, Pyrimidine, Tryptophan, Proline Metabolism Urea cycle	Negative
Glycochenodeoxycholic acid 3- glucuronide	NEG and POS	T0	NA	Negative
LysoPC(18:2/0:0)	POS	TX	NA	Positive
N-Acetyl-L-aspartic acid	NEG	TX	Aspartate Metabolism	Positive
Uridine	NEG and POS	TX	Pyrimidine Metabolism/ Pyrimidine Ribonucleosides Degradation	Positive

*NEG* negative charge, *POS* positive charge, *T0* treatment baseline, *TX* other time point during psychotropic treatment, *HMDB* human metabolome database.

^#^Non-exhaustive list. For the exhaustive list see the HMDB website.

On the other hand, increases in uridine intensity level changes were positively associated with our outcome. Uridine represents one of the most important elements for glycogen synthesis under the form of UDP-glucose [24], and it plays a pivotal role in controlling energy intake by acting in a negative feedback loop, in which fasting conditions and caloric intake can remarkably increase and decrease its blood levels, respectively [25, 26]. Interestingly, uridine supplements were associated with increased weight in a preclinical study [27], and with increased food consumption in two placebo-controlled studies including healthy individuals [28]. Both studies are in line with the present results, showing that increases in uridine intensity levels were associated with an increase in weight change, probably through an increase in food consumption.

Among most abundant NEG metabolites at TX, the increase in the intensity levels of 5-hydroxy-L-tryptophan, 1-methyluric acid, and N-acetyl-L-aspartic acid, were also positively associated with our outcome. Previous preclinical studies showed that the serotonin precursor 5-hydroxy-L-tryptophan can suppress food intake [29, 30], which is in contrast with the present results indicating that higher weight increases are associated with higher increase in the intensity of 5-hydroxy-L-tryptophan levels. On the other hand, other studies indicated that 5-hydroxy-L-tryptophan can increase glucocorticoid hormone response [31, 32], notably among females affected by major depressive disorder [33], which would lead to increased food intake [34], and a recent study highlighted a positive association between 5-hydroxy-L-tryptophan and BMI[35]. Thus, these mixed findings concerning 5-hydroxy-L-tryptophan call for further studies focusing on its role in psychotropic drug-induced weight change.

To the best of our knowledge, only one study conducted among two population-based cohorts showed that 1-methyluric acid, a xanthine metabolite of caffeine, was a protective factor for type II diabetes[36]. Likewise, the effects of N-acetyl-L-aspartic acid, an amino acid mostly found in mammalian brains that contributes to myelin production regulation and protein synthesis, was analyzed only in a few preclinical studies, revealing no effects on weight increase nor food intake[37, 38].

Among the most abundant POS metabolites at baseline, the increase in the intensity level changes of glutamine and 4,8 Dimethylnonanoyl carnitine were negatively associated with psychotropic-induced weight gain. Of note, the amino acid glutamine was previously shown to be downregulated in obesity, contributing to white adipose tissue inflammation [39], and a causal link between elevated BMI and lower glutamine levels was also established in a mendelian randomization analysis[40]. These previous findings are in line with the present results reporting a negative association between weight change and the increase in glutamine intensity levels. Moreover, glutamine was also less abundant after one month of psychotropic treatment (average of treatment duration at TX), suggesting a downregulation induced by weight gain.

Alterations in acylcarnitine levels were not only previously associated with metabolic alterations (e.g., obesity [41]), but also with psychotropic-induced weight gain[9]. Indeed, acylcarnitines play a crucial role in β-oxidation by facilitating the transport of long-chain fatty acids into the mitochondria. Their alterations, possibly due to psychotropic treatment, may suggest mitochondrial dysfunction and impaired fatty acid β-oxidation. A previous study focusing on a targeted metabolomic approach found that a decrease in the acylcarnitine hexanoylcarnitine levels was associated with psychotropic-induced weight gain after three months of treatment[9]. This is in agreement with the present results showing a negative association between the increase in 4,8 Dimethylnonanoyl carnitine intensity levels and weight gain under psychotropic treatment. Moreover, another study conducting an untargeted metabolomic analysis among 27 females receiving the weight-inducing psychotropic drug olanzapine showed a decrease in carnitine levels after four weeks of treatment [11], in line with our results reporting more abundant levels of 4,8 Dimethylnonanoyl carnitine at T0 versus TX.

Among the TX-abundant POS metabolites, increases in the intensity level changes of citrulline, the LysoPC(18:2/0:0) and acetylkynurenine were positively associated with our outcome. The nonessential amino acid citrulline is involved in the urea, arginine, and nitric oxide synthesis pathways[42]. Interestingly, citrulline supplementation is often used to increase physical activity performance, which can be followed by a decrease in both fat mass and BMI [43, 44], and lower levels of citrulline were previously found among obese individuals[45]. However our study highlighted that increases in the intensity levels of citrulline were associated with greater weight increase, which is in line with preclinical studies reporting a positive correlation between citrulline and psychotropic drug-induced weight gain in mice [46], and increased citrulline levels in obese mice[47]. Hence, these contrasting results call for further studies on citrulline’s role in the context of psychotropic-induced weight change.

In agreement with our results reporting a positive association between the increase in LysoPC(18:2/0:0) intensity levels and our outcome, several LysoPCs were previously positively associated with olanzapine-induced weight gain in an untargeted metabolomic analysis[11]. Of note, LysoPCs and other glycerophospholipids such as phosphatidylcholines, phosphatidylethanolamines and phosphatidylinositols play a key role in cell membrane structure and function, lipid metabolism and cellular signaling. Interestingly, although identified with a level III degree of confidence, the changes in the intensity levels of other glycerophospholipids (e.g., phosphoetanolamines, phosphatidylcholines) were also found to be associated with our outcome in the present study. Thus, our results further highlight that LysoPCs and glycerophospholipid dysregulation could be used as a fingerprint of psychotropic drug-induced weight gain. Tryptophan metabolites such as kynurenine were previously associated with psychotropic drug-induced weight gain and psychiatric disorders[9, 48]. In the present study, weight change was positively associated with an increase in acetylkynurenine intensity levels, a potential metabolite of acetyltryptophan [49], and, although with minor consistence, with an increase in the intensity levels of kynurenine in our one-iteration LASSO analysis. To the best of our knowledge, the exact role of acetylkynurenine in metabolic pathways still needs to be disclosed, calling for further studies to better understand its involvement in psychotropic drug-induced weight increase. However, our results further support the hypothesis that tryptophan metabolites are involved in metabolic impairments under psychotropic treatment. Interestingly, among POS results, the increase in the intensity levels of one metabolite of the alkaloid aglycone solanidine [50] was also positively associated with our outcome. Solanidine is commonly found in potatoes (*Solanum tuberosum*), and it has been recently considered a ubiquitous marker of cytochrome P450 2D6 activity[50]. Hence, given the ubiquitous nature of solanidine and considering previous studies indicating that high-caloric food consumption (e.g., French fries, chips) is highly prevalent among patients receiving psychotropic drugs [51, 52], the present result suggests that solanidine metabolite association with weight gain could reflect dietary habits among our psychiatric cohort.

Several limitations must be mentioned. First, we could not control for lifestyle differences (i.e., dietary or physical activity differences) between patients and between T0 and TX, nor for pathological conditions (e.g., non-psychiatric diseases) or environmental exposures (e.g., drug and alcohol consumption). These confounding factors should be addressed in further studies, as they are likely to influence both metabolomic results (e.g., through variations in the gut microbiome) and weight change. Second, although we could benefit from a greater sample size than previous studies [9, 11], we could not analyze the specific metabolomic fingerprint of each psychotropic drug (e.g., the metabolomic fingerprint of the antipsychotic quetiapine or of the mood stabiliser lithium separately) because of the low number of patients when considering each drug separately. As different metabolic signatures could be expected, further studies should focus on identifying single-drug metabolomic changes to better elucidate the different mechanisms associated with psychotropic-induced weight gain. Third, whether patients were psychotropic drug-naïve or were previously treated was also unknown, although, given the overall median age of 43 years, we are confident that most of the included individuals had already taken psychotropic drugs prior to this study, reflecting most of the psychiatric population in naturalistic studies. On the other hand, one of the main strengths of the present study was the machine learning approach. Indeed, our LASSO bootstraps selected only the most stable metabolites associated with our outcome by creating 5000 sub-cohorts from our 151 follow-ups (i.e., by performing 5000 separate LASSO metabolite selection models). Moreover, thanks to the longitudinal approach, each participant was their own control. Hence, this study design allowed us to confidently describe changes in the metabolomic fingerprint, suggesting novel biomarkers and metabolic pathways linked with weight change under psychotropic treatment. Future studies should validate these biomarkers and explore their potential for preventing or mitigating psychotropic-induced weight gain. More specifically, targeted quantitative validation of key metabolites would be required to confirm absolute concentration changes and to further strengthen the translational relevance of these findings.

In conclusion, the present study highlighted novel biomarkers for weight change under psychotropic treatment, and it confirmed the involvement of glycerophospholipids and tryptophan metabolites. These results emphasise the potential use of metabolomics analysis and machine learning approaches combined, not only to further elucidate metabolic pathways, but also to identify biomarkers that could play a role in guiding early metabolic monitoring and in personalizing strategies aimed at preventing or reducing important weight gain.

### Data sharing

Data from PsyMetab cannot be publicly deposited due to participant confidentiality purposes. Data from PsyMetab can be accessed after formal application and ethical review by the Ethics Committee of the Canton of Vaud. For further details: http://www.chuv.ch/cnp-psymetab. The scripts used to produce the results are available on: https://github.com/ranjbar/Metabolomic-Feature-Selection

## Supplementary information

Online supplement
