# Supplementary material for: Psychotropic drug-induced weight gain and untargeted metabolomics: machine learning-driven results from a prospective cohort study
Source: Transl Psychiatry. 2026 Jun 27;16:467. doi: 10.1038/s41398-026-04129-9 (PMC13572460; doi:10.1038/s41398-026-04129-9)
Supplement: Supplementary file 1 [file 41398_2026_4129_MOESM1_ESM.pdf]

Online supplement.

Supplementary Table 1. List of co-medications targeting metabolic and psychiatric disorders considered in the present study.

Supplementary Table 2. Algorithm for metabolic feature selection.

Supplementary Table 3. 100 POS and NEG mRMR selected features.

Supplementary Table 4. Selected features using mRMR and LASSO with 5000 bootstraps.

Supplementary Table 5. Identification of metabolites selected with one LASSO iteration according to Metabolomics Standards Initiative criteria.

Supplementary Figure 1. Minimum Redundancy Maximum Relevance threshold choice.

Supplementary Figure 2. Flowchart of metabolic features selection.

Supplementary Figure 3. Principal component analysis score plots before and after LOWESS normalization.

Supplementary Figure 4. Selection consistency of metabolites in LASSO Bootstrap Analysis.

Supplementary Figure 5. Intensity changes versus weight change for positively charged metabolites selected with Least Absolute Shrinkage and Selection Operator (LASSO).

Supplementary Figure 6. Intensity changes versus weight change for negatively charged metabolites selected with Least Absolute Shrinkage and Selection Operator (LASSO).

Supplementary Table 1. List of co-medications targeting metabolic and psychiatric disorders considered in the present study.

**Weight-inducing psychotropic drugs**	**Lipid-lowering drugs**	**Antidiabetic drugs**	**Antihypertensive drugs**
Amisulpride	Acipimox	Acabrose	Amlodipine
Amitriptyline	Bezafibrate	Albiglutide	Atenolol
Aripiprazole	Alirocumab	Alogliptin	Azilsartan medoxomil
Asenapine	Evolocumab	Canagliflozin	Benazepril
Brexpiprazole	Colestipol	Dulaglutide	Bisoprolol
Carbamazepine	Colextran	Empagliflozin	Candesartan
Cariprazine	Colesevelam	Ertugliflozin	Captopril
Chlorprotixene	Colestyramine	Exenatide	Carvedilol
Clomipramine	Atorvastatin	Glibenclamide	Celiprolol
Dibenzepine	Choline	Glibornuride	Clonidine
Doxepine	Fenofibrate	Gliclazide	Diltiazem
Flupentixol	Gemfibrozil	Glimepiride	Doxazosin
Fluphenazine	Ezetimibe	Insulin	Enalapril
Haloperidol	Fenofibrate	Linagliptin	Eprosartan
Imipramine	Fluvastatin	Liraglutide	Esmolol
Levomepromazine	Pitavastatin	Lixisenatide	Felodipine
Lithium	Pravastatin	Semaglutide	Furasemide
Lurasidone	Rosuvastatin	Metformin	Glyceryl trinitrate
Mirtazapine	Simvastatin	Nateglinide	Hydrochlorothiazide
Nortriptyline		Pioglitazone	Indapamide
Olanzapine		Repaglinide	Irbesartan
Opipramol		Saxagliptin	Isradipine
Paliperidone		Sitagliptin	Labetalol
Pipamperone		Vildagliptin	Lercanidipine
Promazine			Lisinopril
Quetiapine			Losartan
Risperidone			Metoprolol
Sertindole			Minoxidil
Sulpiride			Moxonidine
Tiapride			Nebivolol
Trimipramine			Nifedipine
Valproate			Nitrendipine
Zuclopenthixol			Olmesartan medoxomil
			Perindopril
			Quinapril
			Ramipril
			Spironolactone
			Telmisartan
			Torasemide
			Urapidil
			Valsartan
			Verapamil

Supplementary Table 2. Algorithm for metabolic feature selection.

**Algorithm : Metabolic Feature Selection**	
**Step 1 :**	**Quality Control (QC) Filtering**<br>*Input:* $\mathcal{D}_{pos}, \mathcal{D}_{neg}$ Datasets of positive and negative metabolic features' QC, $CV_{threshold} = 20\%$<br>For each metabolic feature $m_i \in \mathcal{D}_{pos} \cup \mathcal{D}_{neg}$:<br>• Compute the coefficient of variation $CV(m_i) = \frac{\sigma(m_i)}{\mu(m_i)} \times 100$<br>• If $CV(m_i) > CV_{threshold}$, eliminate m_i.<br>*Output :*<br>• $\mathcal{D}_{pos}^1 = \{m_i \in \mathcal{D}_{pos} \mid CV(m_i) \leq 20\% \}$<br>• $\mathcal{D}_{neg}^1 = \{m_i \in \mathcal{D}_{neg} \mid CV(m_i) \leq 20\% \}$
**Step 2 :**	**Minimum Redundancy Maximum Relevance (mRMR) Selection**<br>*Input:* $\mathcal{D}_{pos}^1, \mathcal{D}_{neg}^1$, K=100: Maximum number feature to select via mRMR<br>Define $\Delta I(m_i)$ as the monthly intensity change for each metabolic feature and Y as the average monthly weight change.<br>For each *iteration* $k \in \{1,2,\dots,K\}$:<br>• For each remaining feature $m_i \in \mathcal{D}_{pos}^1 \; or \; \mathcal{D}_{neg}^1$, compute the Spearman Correlation Quotient (SCQ):<br>$$SCQ(m_i) = \frac{I(Y; \Delta I(m_i))}{\frac{1}{k-1} \sum_{m_j \in S} I\left(\Delta \mathrm{I}(\mathrm{m_j}); \Delta \mathrm{I}(\mathrm{m_i})\right)}$$<br>Where $I(X;Y)$ denotes the mutual information between X and Y, and S is the set of metabolic features selected in previous iterations.<br>• Select m_i such that:<br>$$m_i = \arg \max_{m \in \mathcal{D}_{pos}^1 \cup \mathcal{D}_{neg}^1} SCQ(m)$$<br>• Add m_i to the set of selected features $S_{pos} \; or \; S_{neg}$.<br><br>*Output:*<br>• $S_{pos} \subseteq \mathcal{D}_{pos}^1$ such that $\lvert S_{pos} \rvert = 100$<br>• $S_{neg} \subseteq \mathcal{D}_{neg}^1$ such that $\lvert S_{neg} \rvert = 100$
**Step 3 :**	**LASSO Selection with Bootstrapping**<br>*Input:* S_{pos}, S_{neg}, $B = 5000$: Number of bootstrap iteration, $F = 10$: Number of cross-validation folds.<br>For each bootstrap iteration $b \in \{1,2,\dots,B\}$:<br>• Randomly sample with replacement from the datasets S_{pos} and S_{neg}, separately.<br>• Apply 10-fold cross-validation to select the optimal regularization parameter λ_b for LASSO:<br>$$\hat{\beta}^b = \arg \min_{\beta} \left(\frac{1}{n} \sum_{i=1}^{n} (Y_i - X_i^T \beta)^2 + \lambda_b \lVert \beta \rVert_1 \right)$$<br>• Record the selected features $S^{(b)}$ and their respective signs.<br>Stability check:<br>• For each metabolic feature $m_i \in S_{pos} \cup S_{neg}$, calculate its selection frequency:<br>$$f(m_i) = \frac{1}{B} \sum_{b=1}^{B} I(m_i \in S^b) \times 100$$<br>• Retain m_i if: $f(m_i) \geq 50\%$ and sign of β_i is stable within the 95% empirical confidence interval.<br>*Output:* Final selected metabolic features $\hat{S}_{pos} \; and \; \hat{S}_{neg}$ with the stable sign and high frequency.
**End Algorithm**	

Supplementary Table 3. 100 POS and NEG mRMR selected features.

m/z NEG	RT (s)	Score mRMR	m/z POS	RT (s)	Score mRMR
**313.1196**	**6.0**	**0.0718**	**341.1703**	**3.1**	**0.0412**
**309.1112**	**5.2**	**0.0466**	**147.0761**	**0.4**	**0.0339**
**328.8943**	**0.3**	**0.0409**	**176.1028**	**0.4**	**0.0337**
**349.1111**	**5.4**	**0.0368**	**915.5219**	**6.5**	**0.0316**
**273.1709**	**5.7**	**0.0352**	**251.1022**	**3.5**	**0.0307**
**376.2704**	**6.0**	**0.0304**	**267.0583**	**0.7**	**0.0306**
**397.1685**	**5.8**	**0.0294**	**626.3522**	**5.6**	**0.0304**
**550.3149**	**6.4**	**0.0282**	**187.0575**	**0.4**	**0.0297**
**289.0680**	**0.7**	**0.0252**	**303.6463**	**7.0**	**0.0295**
**230.9000**	**0.4**	**0.0246**	**770.5676**	**9.0**	**0.0293**
**846.5486**	**9.3**	**0.0220**	**722.8872**	**0.3**	**0.0291**
**227.0023**	**4.3**	**0.0217**	**204.0993**	**5.4**	**0.0266**
**588.6821**	**0.4**	**0.0212**	**311.1849**	**5.8**	**0.0264**
**302.7461**	**0.4**	**0.0197**	**585.2009**	**5.0**	**0.0249**
**281.0361**	**0.7**	**0.0197**	**396.0854**	**5.2**	**0.0234**
**345.2064**	**7.0**	**0.0195**	**155.1541**	**2.5**	**0.0228**
**181.0366**	**1.3**	**0.0195**	**193.1585**	**6.1**	**0.022**
**219.0775**	**0.9**	**0.0188**	**303.1437**	**5.0**	**0.0208**
**870.8044**	**0.3**	**0.0187**	**129.0656**	**0.4**	**0.0207**
**624.3387**	**5.6**	**0.0182**	**198.0848**	**0.4**	**0.0195**
**519.1511**	**4.4**	**0.0179**	**209.1284**	**4.0**	**0.019**
**250.8335**	**0.4**	**0.0179**	**542.3214**	**7.0**	**0.0184**
**731.0413**	**0.4**	**0.0171**	**868.6180**	**9.8**	**0.0183**
**620.3550**	**7.4**	**0.0168**	**416.3158**	**4.3**	**0.017**
**521.2761**	**6.5**	**0.0158**	**224.2005**	**6.0**	**0.0169**
**174.0410**	**0.4**	**0.0158**	**330.2636**	**5.8**	**0.0168**
**319.1316**	**6.1**	**0.0156**	**700.5270**	**9.2**	**0.0167**
**828.5763**	**9.2**	**0.0155**	**632.7983**	**0.3**	**0.0167**
301.0211	*0.7*	*0.0494*	*525.3297*	*6.0*	*0.0374*
146.0460	*0.4*	*0.0309*	*722.5111*	*8.9*	*0.0275*
583.3124	*5.8*	*0.0301*	*338.1344*	*2.8*	*0.0231*
565.3018	*5.6*	*0.0288*	*418.0733*	*4.0*	*0.0215*
313.2384	*7.3*	*0.0271*	*545.0992*	*1.1*	*0.0207*
511.2914	*5.3*	*0.0269*	*463.2684*	*6.1*	*0.0204*
240.9540	*0.6*	*0.0269*	*223.1690*	*5.5*	*0.0196*
606.3285	*5.9*	*0.0256*	*236.9979*	*0.6*	*0.0192*
459.1505	*5.7*	*0.0254*	*486.7853*	*0.4*	*0.0185*
319.1318	*5.9*	*0.0251*	*247.1282*	*1.2*	*0.0185*
262.0255	*3.8*	*0.0239*	*367.2086*	*6.3*	*0.0176*
495.2597	*5.9*	*0.0217*	*213.1230*	*2.6*	*0.0172*
665.1818	*2.4*	*0.0211*	*209.0915*	*1.1*	*0.0169*
549.3070	*6.7*	*0.0206*	*591.3170*	*6.6*	*0.0169*

375.1310	*3.7*	*0.0202*	832.5836	9.0	0.1302
142.0664	*2.7*	*0.0194*	174.0547	1.1	0.0475
778.4516	*6.2*	*0.0194*	477.2845	5.3	0.0375
481.1874	*6.1*	*0.0191*	269.0786	3.9	0.0344
387.2535	*7.1*	*0.0188*	315.1332	6.0	0.0339
341.0670	*3.5*	*0.0186*	234.8933	0.4	0.0327
254.9816	*0.5*	*0.0186*	754.5740	9.9	0.0327
319.1318	*6.0*	*0.0186*	337.1232	3.2	0.0308
272.9576	*0.6*	*0.0185*	207.1742	6.2	0.03
640.3630	*7.2*	*0.0176*	554.0934	1.1	0.0293
222.0773	*2.8*	*0.0175*	485.3838	7.5	0.0292
646.3212	*6.1*	*0.0171*	606.4088	5.9	0.0291
248.0904	*5.4*	*0.0167*	354.8947	0.4	0.0275
186.0562	*3.8*	*0.0161*	834.5977	9.8	0.0275
507.1484	*4.5*	*0.0160*	221.0919	0.9	0.0265
963.1927	*0.4*	*0.0157*	213.1596	4.5	0.0238
337.1422	5.1	0.1432	247.1283	1.4	0.0234
385.1689	5.6	0.0368	710.5103	8.9	0.0233
840.5761	9.2	0.0328	399.2499	6.7	0.0233
245.0935	3.9	0.0325	358.8424	0.4	0.023
190.0509	1.1	0.0320	354.2611	6.0	0.0226
876.5768	8.9	0.0314	855.0845	9.7	0.0224
341.0293	0.7	0.0306	346.1548	2.4	0.022
808.5502	8.9	0.0270	579.3524	6.1	0.0217
293.1161	5.7	0.0265	752.5206	8.4	0.0217
260.1505	3.5	0.0264	442.2911	2.6	0.0215
433.2948	7.3	0.0245	552.3290	6.4	0.0214
270.8908	0.3	0.0244	412.2840	5.6	0.0213
183.1029	5.5	0.0222	247.1075	3.9	0.0212
195.0510	0.4	0.0218	939.6398	5.7	0.0209
602.3019	7.0	0.0217	744.5894	9.3	0.0208
387.1841	5.4	0.0215	726.5416	9.4	0.0207
207.0775	1.1	0.0206	339.1042	3.6	0.0206
856.6081	9.8	0.0204	674.5712	7.4	0.0205
321.1473	6.2	0.0203	752.9003	0.3	0.0205
337.1422	5.5	0.0201	189.1345	0.4	0.0203
280.0971	4.7	0.0200	443.0047	0.3	0.0193
191.0197	0.6	0.0200	207.1740	6.1	0.0192
366.2415	5.9	0.0199	163.0864	1.1	0.0192
461.2903	6.2	0.0196	207.0140	0.3	0.0191
482.1403	2.9	0.0194	862.6291	10.2	0.019
535.3126	6.2	0.0188	643.3796	6.1	0.019
291.1369	4.3	0.0186	470.3837	7.3	0.0189
164.0353	3.9	0.0185	243.1952	6.1	0.0188
541.2655	5.3	0.0181	283.0323	0.7	0.0185

295.1320	5.8	0.0176	195.1126	3.3	0.0183
387.1840	5.4	0.0173	425.7846	9.7	0.0182
377.1814	4.9	0.0173	466.3160	5.8	0.0182
360.7941	0.4	0.0173	247.0850	4.4	0.0179
267.2331	6.8	0.0171	290.1592	1.8	0.0179
455.0055	0.3	0.0167	774.5036	8.4	0.0178
983.5416	6.1	0.0165	281.1129	2.7	0.017
203.0206	0.4	0.0165	648.3345	5.6	0.017
563.3229	6.6	0.0164	812.6154	9.8	0.0169
243.0635	0.7	0.0163	590.3214	7.0	0.0168
342.2284	6.4	0.0162	416.8009	0.4	0.0168
512.9647	0.3	0.0159	937.5019	5.7	0.0167
302.6606	5.9	0.0159	242.2111	6.1	0.0166

Metabolites in bold were selected after applying 5000 Least Absolute Shrinkage and Selection Operator (LASSO). Metabolites in italic blue were selected after applying 1xLASSO. Of note, all metabolites selected with 5000 LASSO were also included after applying one LASSO iteration only. The other metabolites (i.e., neither bold, nor blue) were included in the 100 selected metabolites through the mRMR algorithm.
Abbreviations: RT: retention time; m/z: mass over ion charge; mRMR: minimum Redundancy Maximum Relevance.

Supplementary Table 4. Selected features using mRMR and LASSO with 5000 bootstraps.

M/Z	RT	Median delta intensity	Score mRMR	NEG/POS	Most abundant	% BOOTSTRAP selection
328.8943	0.3	1147.95	0.041	NEG	Tx	96
731.0413	0.4	-7603.79	0.017	NEG	T0	93
397.1685	5.8	-19392.50	0.029	NEG	T0	88
828.5763	9.2	577373.33	0.015	NEG	Tx	83
250.8335	0.4	2617.70	0.018	NEG	Tx	83
376.2704	6.0	7607.47	0.030	NEG	Tx	81
345.2064	7.0	-5012.33	0.020	NEG	T0	80
521.2761	6.5	-17052.73	0.016	NEG	T0	79
319.1316	6.1	56337.05	0.016	NEG	Tx	79
313.1196	6.0	-111090.91	0.072	NEG	T0	78
519.1511	4.4	140963.23	0.018	NEG	Tx	78
181.0366	1.3	1051405.68	0.020	NEG	Tx	75
620.3550	7.4	-22617.24	0.017	NEG	T0	74
289.0680	0.7	21360.93	0.025	NEG	Tx	74
846.5486	9.3	-9734.02	0.022	NEG	T0	74
588.6821	0.4	-3486.26	0.021	NEG	T0	73
624.3387	5.6	-37618.90	0.018	NEG	T0	73
302.7461	0.4	2912.76	0.020	NEG	Tx	71
550.3149	6.4	31642.69	0.028	NEG	Tx	71
230.9000	0.4	1083.57	0.025	NEG	Tx	71
349.1111	5.4	13165.12	0.037	NEG	Tx	68
870.8044	0.3	-3408.75	0.019	NEG	T0	65
273.1709	5.7	12007.50	0.035	NEG	Tx	63
219.0775	0.9	7126.67	0.019	NEG	Tx	62
174.0410	0.4	10630.58	0.016	NEG	Tx	61
281.0361	0.7	304777.06	0.020	NEG	Tx	59
227.0023	4.3	75308.30	0.022	NEG	Tx	56
309.1112	5.2	57574.05	0.047	NEG	Tx	54
129.0656	0.4	-226207.50	0.021	POS	T0	95
187.0575	0.4	-470464.24	0.030	POS	T0	94
224.2005	6.0	-1196.75	0.017	POS	T0	94
176.1028	0.4	54459.62	0.034	POS	Tx	85
915.5219	6.5	234320.62	0.032	POS	Tx	85
311.1849	5.8	-51650.00	0.026	POS	T0	83
722.8872	0.3	2242.00	0.029	POS	Tx	79
626.3522	5.6	-20999.43	0.030	POS	T0	72
416.3158	4.3	764.90	0.017	POS	Tx	72
155.1541	2.5	-296351.02	0.023	POS	T0	70
198.0848	0.4	57511.50	0.020	POS	Tx	70
632.7983	0.3	4025.69	0.017	POS	Tx	69
330.2636	5.8	-25549.28	0.017	POS	T0	67
585.2009	5.0	-20318.20	0.025	POS	T0	67

193.1585	6.1	-2620.78	0.022	POS	T0	65
303.1437	5.0	4656.84	0.021	POS	Tx	65
267.0583	0.7	310934.48	0.031	POS	Tx	63
251.1022	3.5	86850.84	0.031	POS	Tx	61
303.6463	7.0	-21877.66	0.030	POS	T0	61
147.0761	0.4	-219428.57	0.034	POS	T0	59
396.0854	5.2	-6857.05	0.023	POS	T0	59
542.3214	7.0	338689.09	0.018	POS	Tx	56
209.1284	4.0	76387.64	0.019	POS	Tx	56
700.5270	9.2	433516.51	0.017	POS	Tx	56
341.1703	3.1	34573.68	0.041	POS	Tx	55
204.0993	5.4	32143.92	0.027	POS	Tx	53
868.6180	9.8	-9372.16	0.018	POS	T0	51
770.5676	9.0	510020.00	0.029	POS	Tx	50

Abbreviations: M:mass; Z:charge; RT: Retention time; NEG: negative charge; POS: positive charge; T0: treatment baseline; TX: other time point during psychotropic treatment

Supplementary Table 5. Identification of metabolites selected with 1xLASSO according to Metabolomics Standards Initiative criteria.

Sign	T	M/Z	RT	Adduct	Chemical formula	Putative name	Putative class	Delta ppm	MSI level	Identifier
NEG	T0	583.3124	5.8	M-H	C30H48O11	Cholic acid glucuronide	Steroid glucuronide conjugates	0.03027537	2*	HMDB0002577
NEG	T0	387.2535	7.1	M-H	C24H36O4	-	Monohydroxy bile acids, alcohols and derivatives		3	
NEG	T0	511.2914	5.3	M-H	C27H44O9	-	Steroidal glycosides		3	
NEG	T0	495.2597	5.9	M-H	C26H40O9	-	Diterpenoids		3	
NEG	T0	313.2384	7.3	M-H	C18H34O4	-	Long-chain fatty acids		3	
NEG	Tx	248.0904	5.4	M-H	C13H15NO4	-	Pyridinolates		3	
NEG	Tx	222.0773	2.8	M-H	C11H13NO4	N-Acetyl-L-tyrosine	Tyrosine and derivatives	0.54980886	1	HMDB0000866
NEG	Tx	778.4516	6.2	M+FA-H	C37H68NO11P	-	Glycerophospholipids		3	
NEG	Tx	375.1310	3.7	M-H	C16H24O10	-	Iridoid O-glycosides		3	
NEG	Tx	254.9816	0.5	M-H	C6H8O9S	-	Sulfated fatty acids		3	
NEG	Tx	146.0460	0.4	M-H	C5H9NO4	Glutamate	Alpha amino acids	0.83692879	1	HMDB0060475
POS	T0	338.1344	2.8	M+H	C15H19N3O6	-	Oligopeptides		3	
POS	T0	525.3297	6.0	M+H	C24H49N2O8P	-	Carbamate esters		3	
POS	TX	418.0733	4.0	M+H	C15H19N3O7S2	-	Peptides		3	
POS	TX	247.1282	1.2	M+H	C10H18N2O5	gamma-Glu-Val	Gamma glutamyl amino acids	-2.63700493	1	HMDB0011172
POS	TX	463.2684	6.1	M+H	C26H38O7	-	Steroid glucuronide conjugates		3	
POS	TX	722.5111	8.9	M+Na	C39H74NO7P	-	Glycerophospholipids		3	
POS	TX	223.1690	5.5	M+H	C14H22O2	-	Long-chain fatty acids		3	
POS	TX	213.1230	2.6	M+H	C10H16N2O3	-	Azoles		3	
POS	TX	209.0915	1.1	M+H	C10H12N2O3	Kynurenine	L-alpha-amino acides	-2.7368326	1	HMDB0000684

Abbreviations: NEG: negative charge; POS: positive charge; T0: treatment baseline; TX: other time point during psychotropic treatment; M: mass; Z: charge.
*Match of MS/MS fragments with deglucuronidated standard.

Supplementary Figure 1. Minimum Redundancy Maximum Relevance threshold choice.

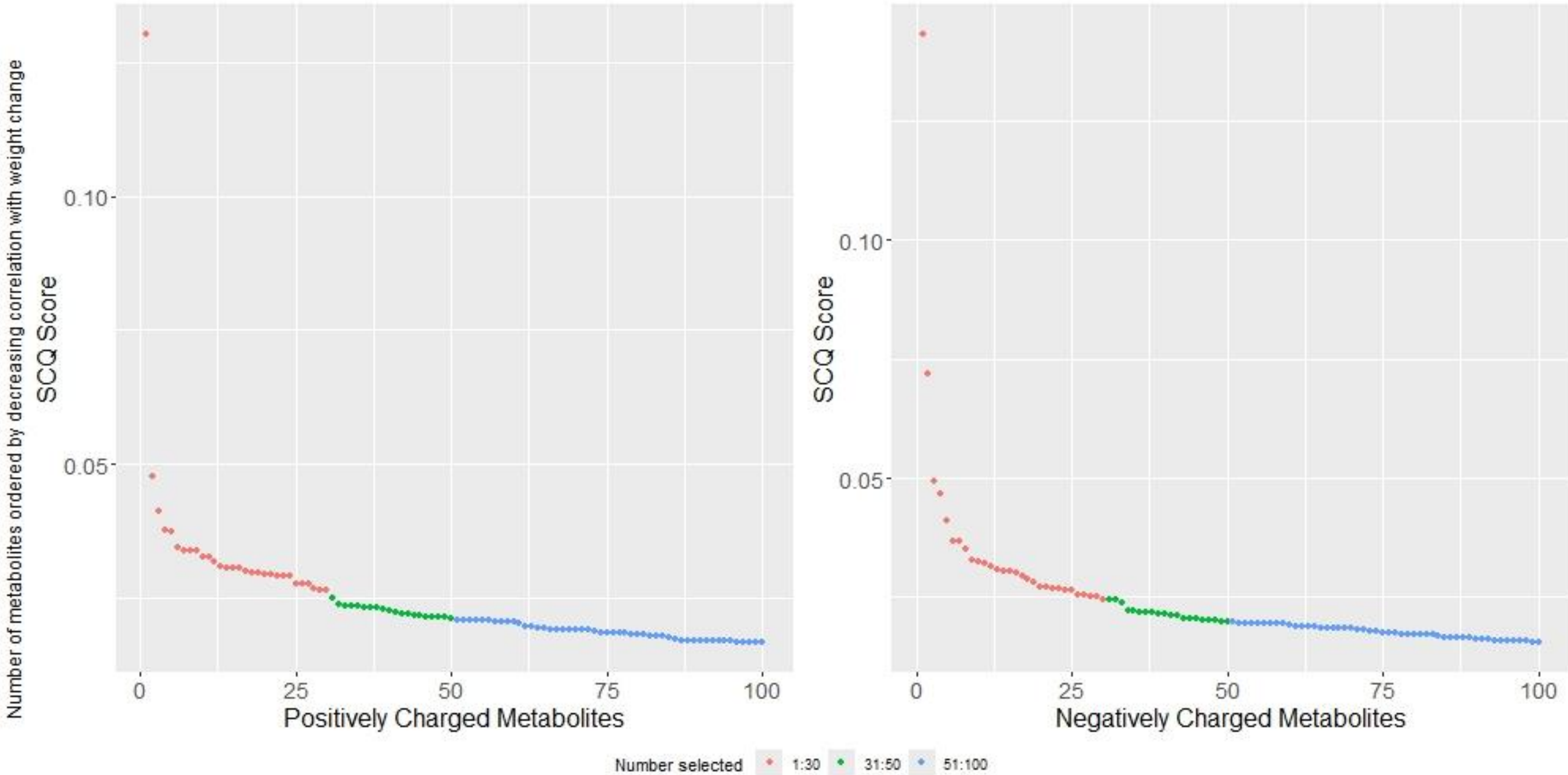

Figure A shows the number of positive metabolites selected according to the Spearman Correlation Quotient (SCQ) Score of the minimum Redundancy Maximum Relevance (mRMR) algorithm.

Figure B shows the number of negative metabolites selected according to the SCQ Score of the mRMR algorithm. This score quantifies the extent to which a selected metabolite is both minimally redundant and maximally relevant with respect to the outcome (i.e., weight change per month of treatment). Higher SCQ values indicate stronger correlations with weight change, and a lower dependency to the already-selected metabolites. Given the plateau in SCQ scores for positive and negative metabolites, no more than 100 metabolites were included in our mRMR algorithm to avoid redundant results.

Supplementary Figure 2. Flowchart of metabolic features selection.

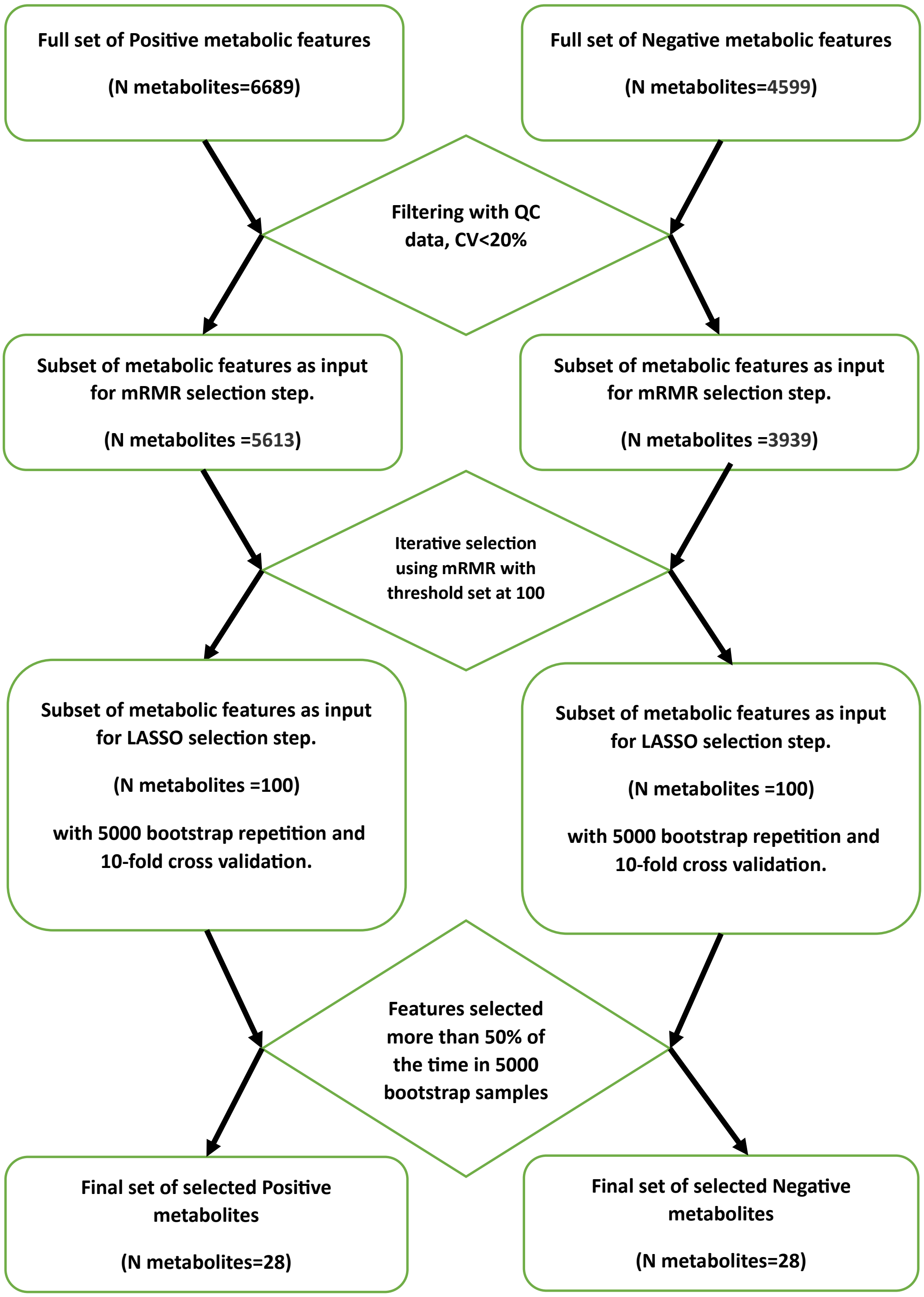

The flowchart resumes the steps used to identify the positively and negatively charged metabolites associated with weight changes. See Methods and Results for further details.

Abbreviations: N: number; QC: quality control; CV: coefficient of variation; mRMR: Minimum Redundancy Maximum Relevance; LASSO: Least Absolute Shrinkage and Selection Operator.

Supplementary Figure 3. Principal component analysis score plots before and after LOWESS normalization.

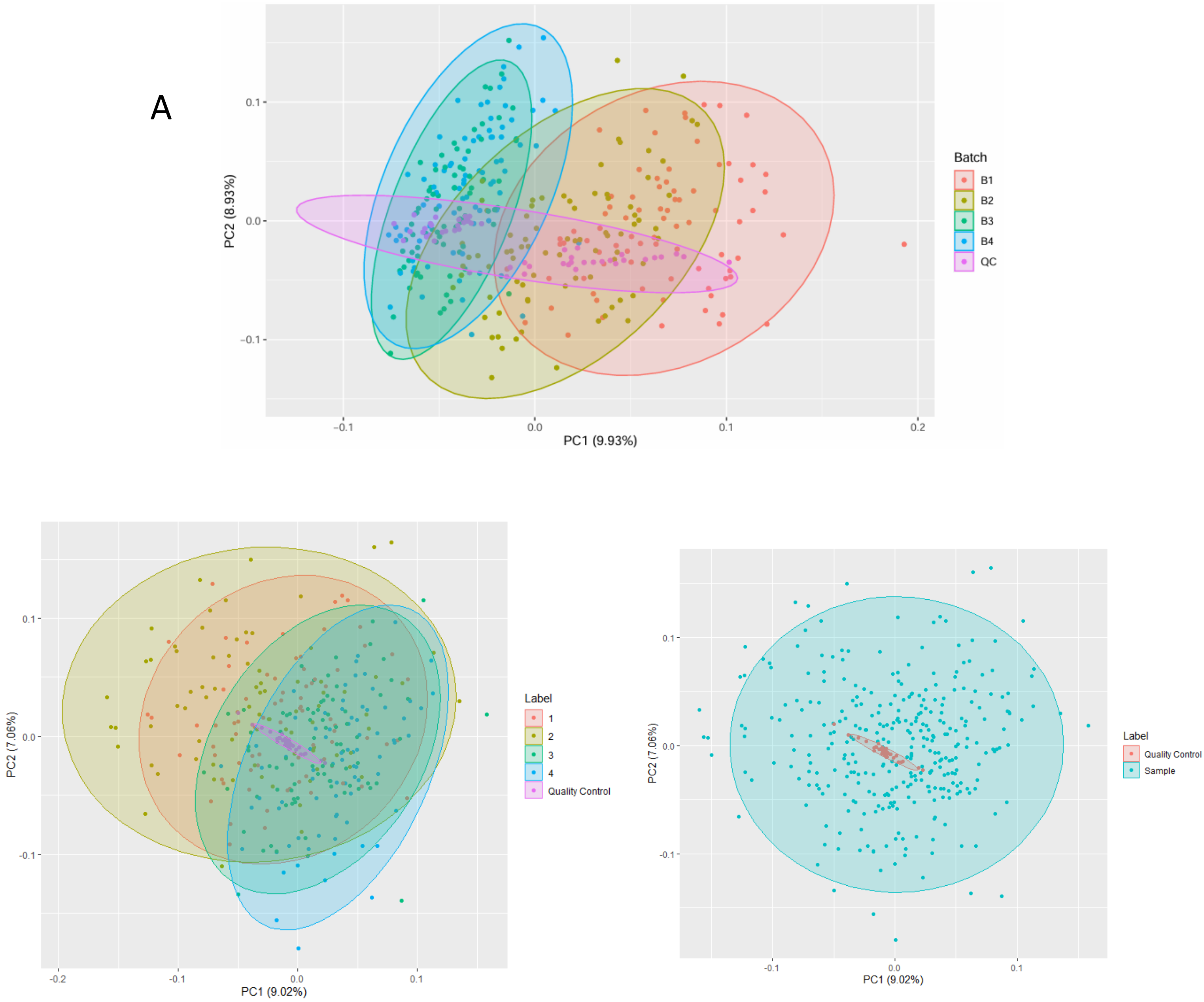

B

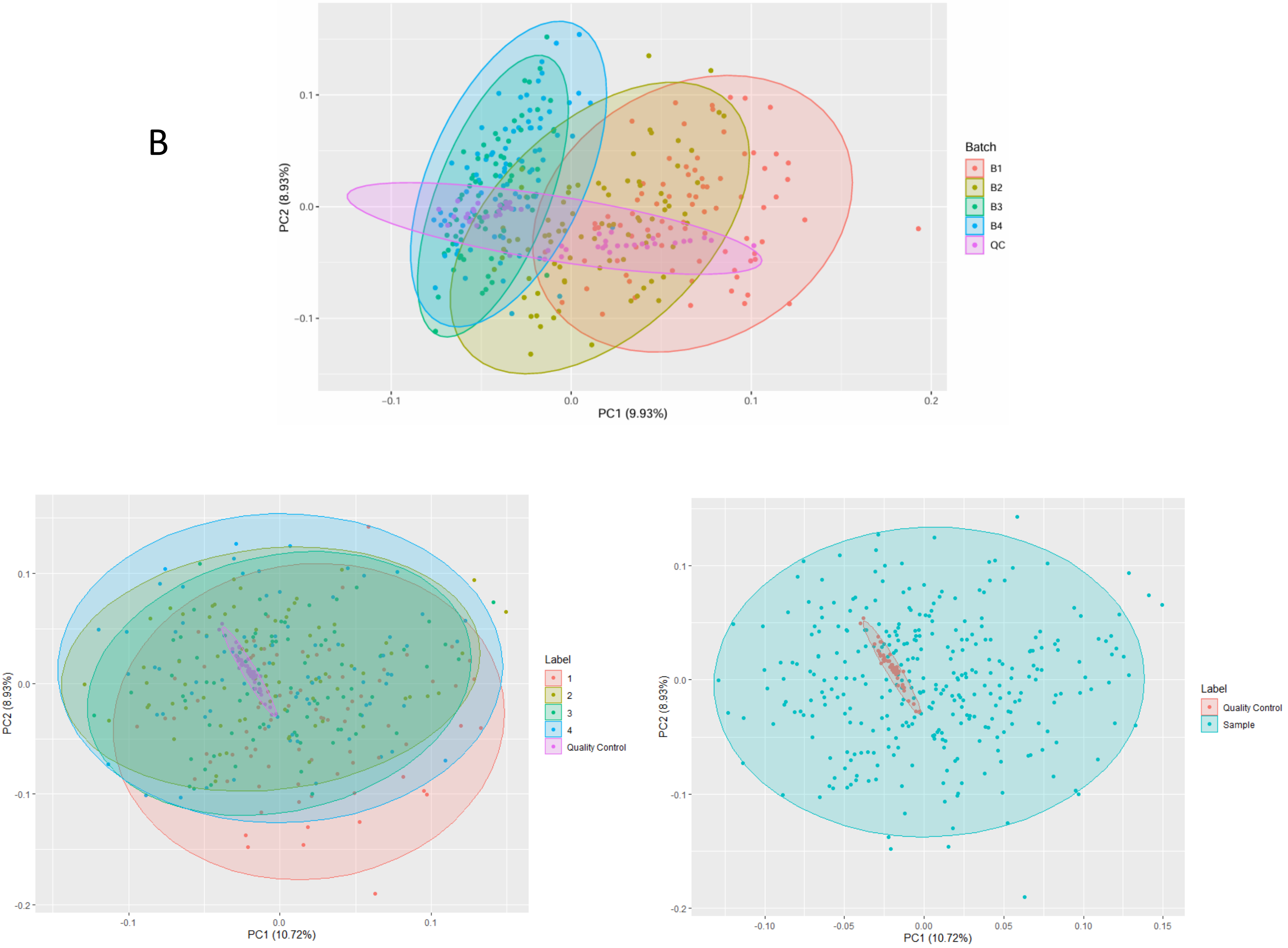

Figures in the A section show the principal component (PC) analysis for the row data (upper figure) and LOWESS-corrected data (lower figures) for the features selected in the positive-mode analysis.
Figures in the B section show the principal component (PC) analysis for the row data (upper figure) and LOWESS-corrected data (lower figures) for the features selected in the negative-mode analysis.
Abbreviations: quality control: QC.

Supplementary Figure 4. Selection consistency of metabolites in LASSO Bootstrap Analysis.

A

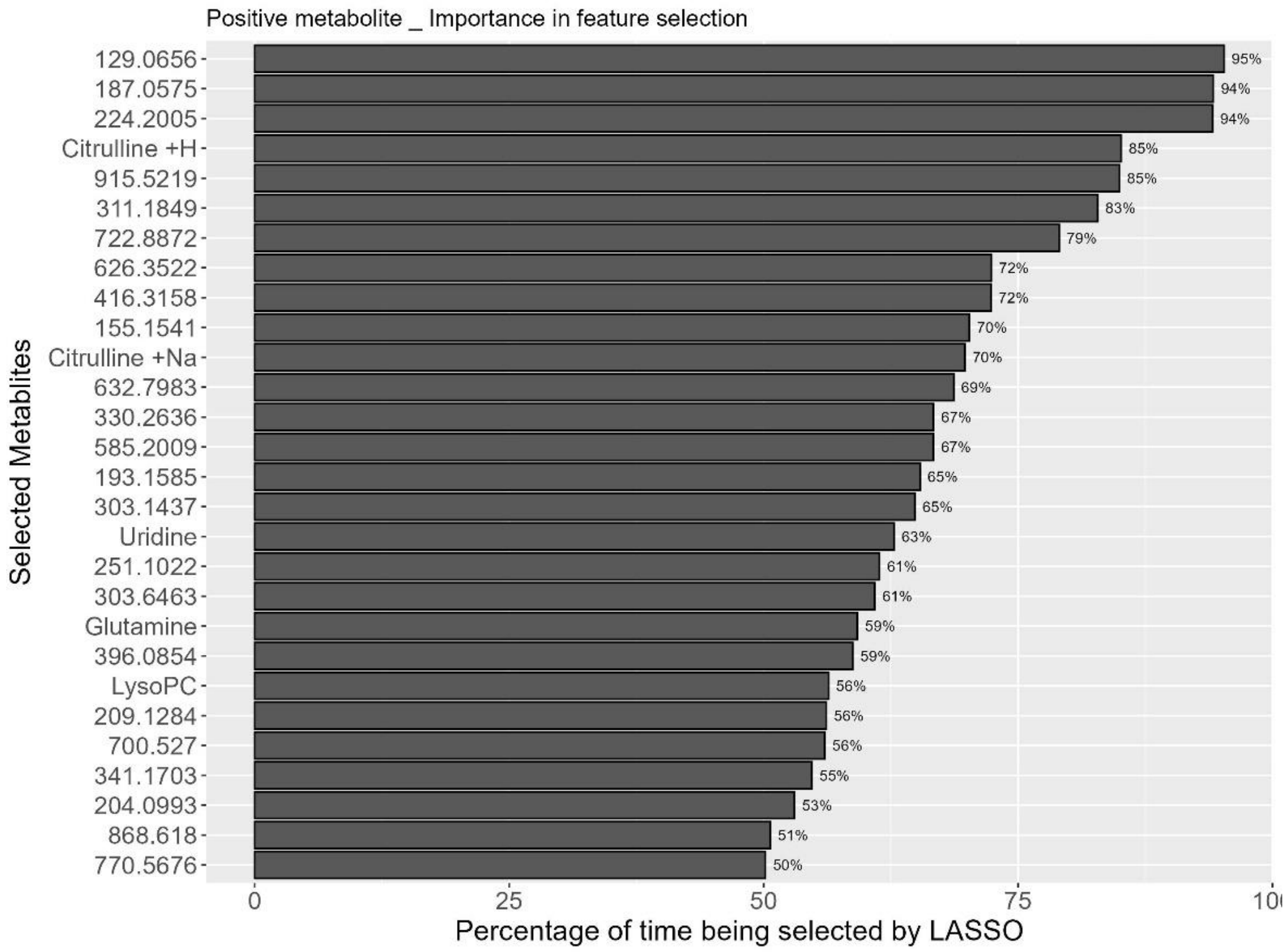

B

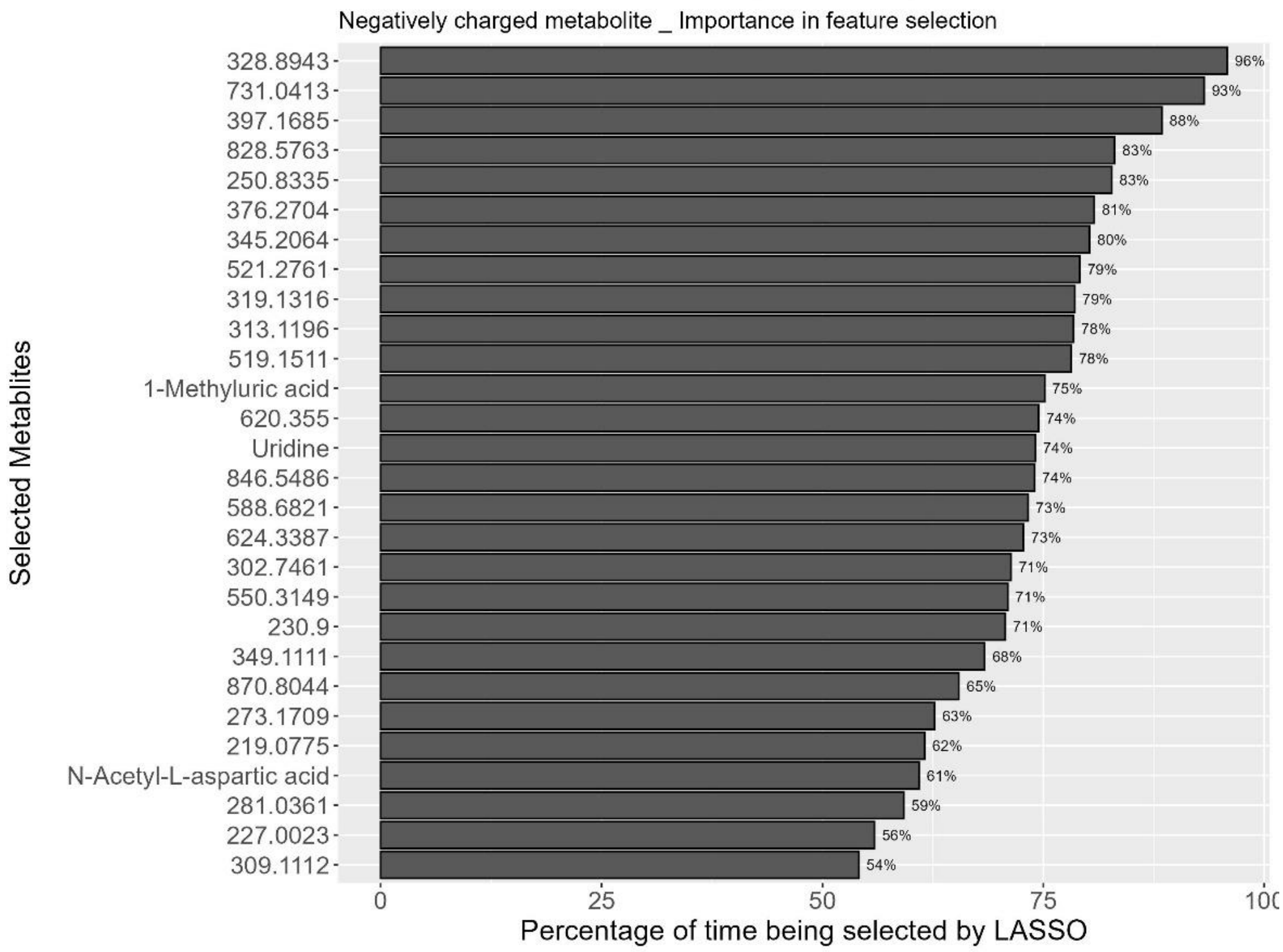

Figure A shows the percentages of times that each negative metabolite was selected with a stable coefficient sign within the 95% empirical confidence interval in the 5000 rounds of Least Absolute Shrinkage and Selection Operator bootstrap. Figure B shows the percentages of times that each positive metabolite was selected with a stable coefficient sign within the 95% empirical confidence interval in the 5000 rounds of Least Absolute Shrinkage and Selection Operator bootstrap.

Supplementary Figure 5. Intensity changes versus weight change for positively charged metabolites selected with Least Absolute Shrinkage and Selection Operator (LASSO).

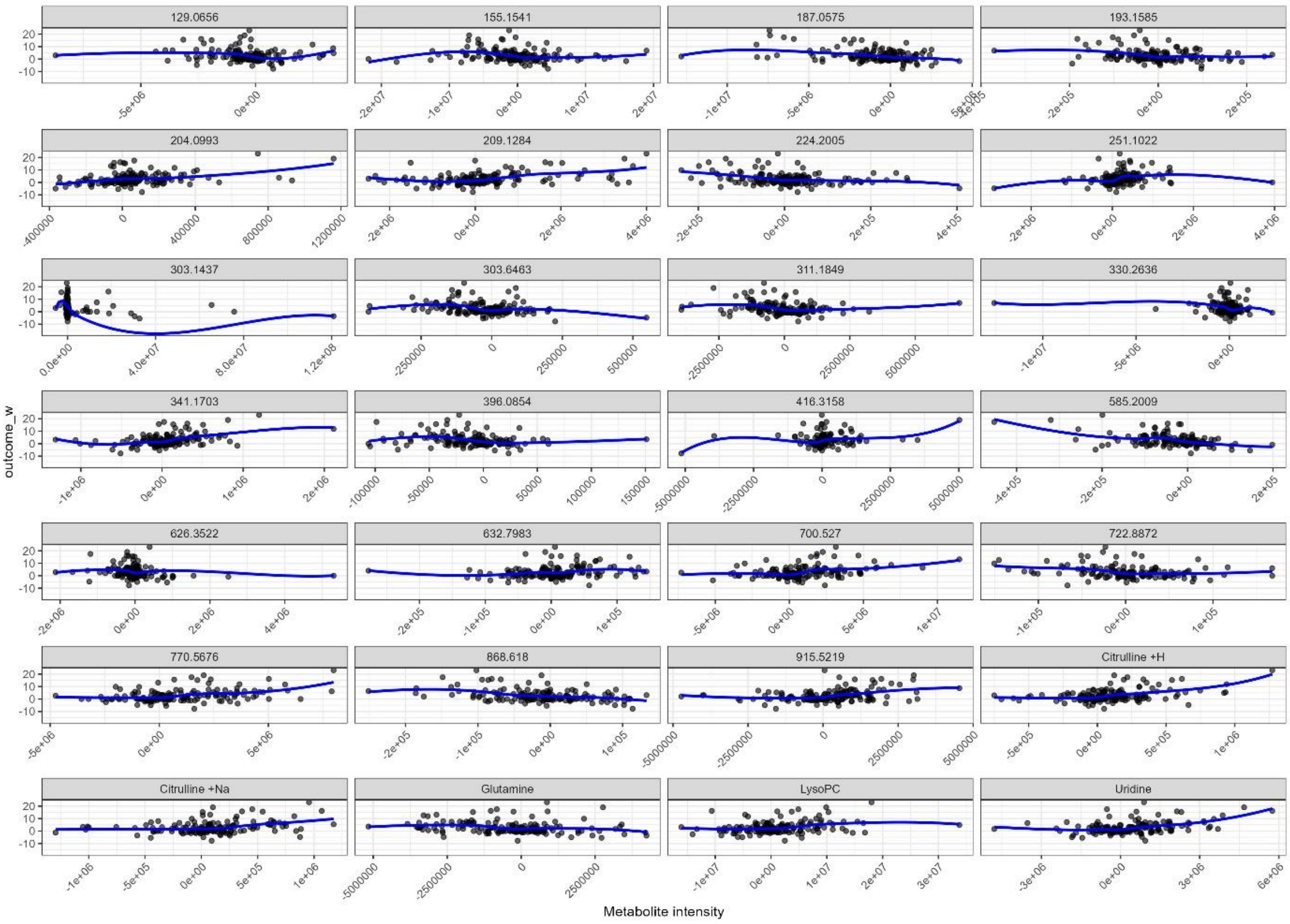

Weight change per month of psychotropic treatment versus intensity change (TX-T0) for positively charged metabolites selected using LASSO. Scatter plot and smoothing spline with 95% confidence bands are reported.

Supplementary Figure 6. Intensity changes versus weight change for negatively charged metabolites selected with Least Absolute Shrinkage and Selection Operator (LASSO).

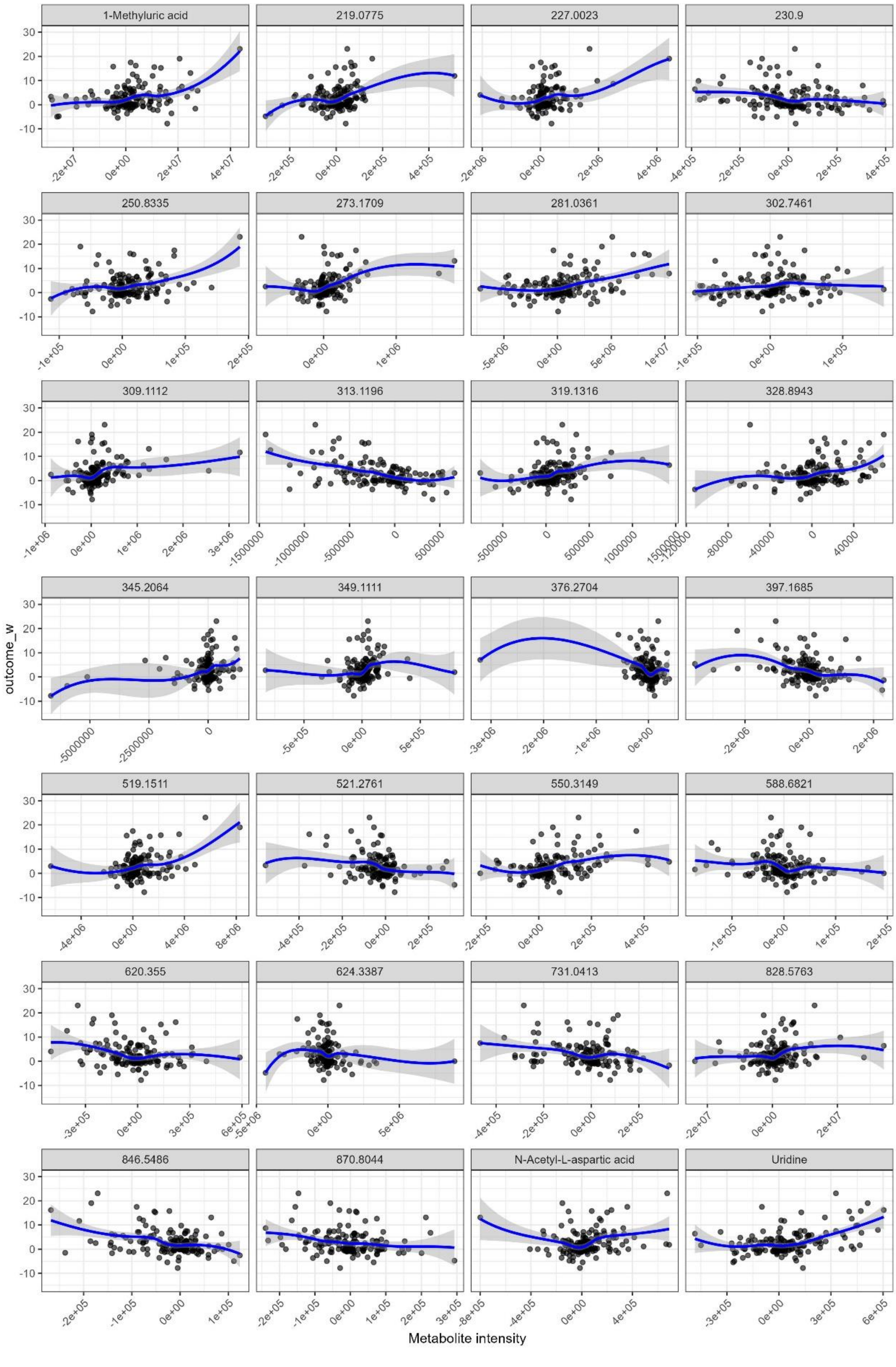

Weight change per month of psychotropic treatment versus intensity change (TX-T0) for positively charged metabolites selected using LASSO. Scatter plot and smoothing spline with 95% confidence bands are reported.
